# Integrated Multi-Omics and Experimental Evidence Implicates PDCD5 as a Candidate Gene Associated with Primary Biliary Cholangitis

**DOI:** 10.3390/life16101583

**Published:** 2026-09-22

**Authors:** Zihao Xu, Yifan Liu, Congcong Cao, Jun Xu

**Affiliations:** 1 School of Chinese Medicine, Hubei University of Chinese Medicine, Wuhan 430065, China; 2527012087@stmail.hbucm.edu.cn (Z.X.); 2232701111@stmail.hbucm.edu.cn (Y.L.); 2 School of Basic Medical Sciences, Hubei University of Chinese Medicine, Wuhan 430065, China

**Keywords:** primary biliary cholangitis, PDCD5, paraptosis, multi-omics, machine learning

## Abstract

Primary biliary cholangitis (PBC) is an autoimmune cholestatic liver disorder in which endoplasmic reticulum stress and mitochondrial dysfunction overlap with features associated with paraptosis. This study aimed to identify a paraptosis-related candidate gene for PBC via multi-omics integration and experimental validation. We integrated four transcriptomic cohorts, constructed a machine learning framework with SHAP analysis, applied two-sample Mendelian randomization (MR), and established a 2-OA-BSA/Poly I:C-induced murine PBC model. The intersection yielded eight candidate genes, and LASSO+RF modeling prioritized four core genes. Genetically predicted PDCD5 and ITPR3 expression was associated with lower PBC odds. In PBC mice, hepatic PDCD5 was downregulated alongside ER dilation, mitochondrial swelling, and stress-related molecular changes. PDCD5 overexpression in human intrahepatic biliary epithelial cells increased metabolic activity measured by CCK-8 under LPS exposure, reduced CHOP, BiP, and p-ERK/ERK, and increased ALIX. These findings nominate PDCD5 as a candidate associated with PBC and provide functional evidence that its overexpression attenuates LPS-associated cellular stress. The specific contribution of paraptosis and the requirement for ERK signaling remain to be established.

## 1. Introduction

Primary biliary cholangitis (PBC) is a chronic autoimmune cholestatic liver disease characterized by progressive, immune-mediated destruction of small intrahepatic bile ducts, potentially progressing to fibrosis, cirrhosis, and end-stage liver failure [1,2]. PBC predominantly affects middle-aged women, with a female to male ratio of approximately 9:1, and its global prevalence has been rising over the past two decades [2]. Ursodeoxycholic acid remains the first-line therapy; however, approximately 30–40% of patients exhibit an incomplete biochemical response, and second-line therapies such as obeticholic acid confer limited efficacy with considerable adverse effects [3]. These treatment limitations highlight the need to better understand PBC pathogenesis and identify therapeutic targets.

The role of programmed cell death in PBC biliary injury has attracted increasing attention. Multiple regulated cell death modalities, including apoptosis, necroptosis, pyroptosis, and ferroptosis, have been implicated in the pathogenesis of hepatobiliary diseases [4]. In PBC specifically, inflammasome-mediated pyroptosis driven by the NLRP3/caspase-1/GSDMD axis has been shown to contribute to cholangiocyte injury and perpetuate the autoimmune inflammatory cascade [5]. Paraptosis is a non-apoptotic form of programmed cell death first described by Sperandio et al. [6,7]. Morphologically, it is distinguished from apoptosis by extensive cytoplasmic vacuolation, endoplasmic reticulum (ER) dilation, and mitochondrial swelling. Biochemically, paraptosis is caspase-independent and has been associated with sustained MAPK/ERK pathway activation [6,7]. In experimental models, paraptosis is inhibited by ALIX (ALG-2-interacting protein X, also known as PDCD6IP) and is mediated by sustained MAPK signaling, including MEK-2, JNK, and ERK [7]. While paraptosis has attracted interest in oncology as a mechanism for overcoming chemoresistance [8,9], its role in autoimmune liver diseases, including PBC, remains unclear.

Mendelian randomization (MR) is an epidemiological approach that uses germline genetic variants as instrumental variables to infer causal relationships between exposures and outcomes, reducing susceptibility to confounding and reverse causation inherent to observational studies [10]. The availability of large-scale cis-expression quantitative trait locus (cis-eQTL) datasets, such as those from the eQTLGen consortium, alongside publicly available genome-wide association study (GWAS) summary statistics, has enabled systematic, high-throughput assessment of gene–disease causal associations via two-sample MR designs [11].

Despite the biological plausibility linking paraptosis to PBC pathogenesis, no study has systematically evaluated the associations between paraptosis-related genes and PBC using a multi-omics MR framework combined with experimental validation. To address this gap, we used a multi-omics framework comprising, firstly, multi-cohort transcriptomic profiling combined with an integrated machine learning pipeline to screen core paraptosis-related candidate genes for PBC; secondly, two-sample MR to evaluate associations between genetically predicted expression of these core genes and PBC; and, finally, in vivo experimental validation to characterize associated molecular and ultrastructural changes in a murine PBC model. We further investigated the effects of PDCD5 overexpression in LPS-treated human intrahepatic biliary epithelial cells.

## 2. Materials and Methods

### 2.1. Identification of Paraptosis-Related Genes

We compiled 66 paraptosis-related genes from the published gene set [12]; the list is provided in Table S1.

### 2.2. Differential Expression Analysis

The public datasets and reference resources used in this study are summarized in Table 1. Transcriptomic data related to PBC were retrieved from the Gene Expression Omnibus (GEO) database: https://www.ncbi.nlm.nih.gov/geo/ (accessed on 10 May 2026). GSE119600 comprised whole-blood samples from 90 PBC patients and 47 controls. GSE93170 comprised peripheral CD4^+^ T-cell samples from six PBC patients and six controls. GSE159676 comprised three PBC liver samples (GSM4837515, GSM4837516, and GSM4837522) and six reference samples (GSM4837490–GSM4837495) obtained from tumor-free liver areas of patients undergoing surgery for colorectal cancer metastases. GSE61260 included 11 PBC and 38 normal control liver samples, selected by the sample-level disease status annotation; other disease categories were excluded. Exact sample identifiers are provided in Table S2. Differential expression was analyzed using limma v3.66.0 with *p* < 0.05. Duplicate gene entries were averaged using avereps. Preprocessing for model construction is described in Section 2.3. The volcano plot was constructed using ggplot2 (version 3.4.4) to illustrate the distribution of DEGs. Bidirectional hierarchical clustering and pheatmap (version 1.0.12) were used to display expression profiles across the PBC and control groups.

### 2.3. Integrated Machine Learning Framework and SHAP Analysis

To identify core candidate genes from the overlap between DEGs and paraptosis-related genes, an integrated machine learning framework was developed. For model construction, expression matrices were restricted to shared genes and jointly adjusted using parametric ComBat (sva), with cohort as the batch variable and no disease status covariate. This adjustment preceded the training/evaluation split. GSE119600 served as the training cohort, while GSE159676, GSE61260, and GSE93170 were used for external evaluation and model selection. Gene expression was subsequently centered and scaled separately in the training cohort and each external cohort. Ten machine learning algorithms—LASSO, Support Vector Machine, Random Forest (RF), GLMBoost, Stepwise GLM, Ridge Regression, Elastic Net, Gradient Boosting Machine, Linear Discriminant Analysis, and XGBoost (version 1.7.6)—were integrated into 113 model combinations. Models retaining three or fewer features were excluded. LASSO used 10-fold internal cross-validation; RF used 1000 trees and nodesize 5. The random seed was 1234. Model performance was compared using AUC across cohorts, and the highest-ranked model was selected. SHapley Additive exPlanations (SHAP) analysis quantified the contribution of each gene feature to model predictions. Code, sample labels, and model parameters are provided in the Supplementary Computational Files.

Using the transcriptomic data from the GSE119600 dataset, box plots were generated with the ggplot2 package in R (version 4.5.2) to compare the normalized expression levels of the identified core genes between the PBC and healthy control groups. Statistical differences between the two groups were evaluated using Student’s *t*-test or Wilcoxon rank-sum test, as appropriate. Additionally, to evaluate the individual discriminative performance of these core genes within the GSE119600 cohort, ROC curves were constructed, and the AUC was calculated using the pROC (version 1.18.5) R package. Internal validation used stratified five-fold outer cross-validation repeated 20 times and five-fold inner cross-validation for LASSO lambda selection (lambda.min; binomial family; AUC criterion). Within each outer training fold, expression was standardized, LASSO-selected features were used to fit RF with 1000 trees and nodesize 5, and predictions were made for the held-out fold. The base seed was 20260826. Participant-level predictions were averaged across repeats to calculate AUC with a DeLong 95% CI, Brier score, calibration slope, and Youden-derived sensitivity and specificity. The initial eight-gene screen preceded this analysis; these metrics therefore evaluate the downstream model conditional on that candidate set.

### 2.4. Data Sources for Mendelian Randomization

The eQTL data for all genes in this study were downloaded from the eQTLGen database: https://www.eqtlgen.org/cis-eqtls.html (accessed on 10 May 2026). All data were cis-eQTLs, derived from blood samples [13]. We screened instrumental variables (SNPs) using the following parameters: *p*-value < 5 × 10^−8^, clumping window: 10,000 kb, r2 < 0.01. For all genes, MR odds ratios were expressed per one-unit increase in genetically predicted gene expression on the effect scale of the eQTLGen-derived exposure data. SNP-level information and sensitivity results are provided in Tables S3 and S4.

PBC GWAS summary statistics (GCST90044188) were downloaded from the GWAS Catalog database: https://www.ebi.ac.uk/gwas/ (accessed on 10 May 2026) [14]. The outcome cohort comprised 121 PBC cases and 456,227 controls of European ancestry from UK Biobank (Table 1). Exposure–outcome sample overlap was not quantified.

### 2.5. Two-Sample MR Analysis

MR interpretation relies on the three core instrumental variable assumptions: (1) relevance assumption: genetic variants must be robustly associated with the exposure; (2) independence assumption: variants must be independent of any unmeasured confounders influencing the exposure–outcome pathway; and (3) exclusion restriction: variants must affect the risk of PBC exclusively via the exposure rather than other pleiotropic pathways. Regarding missing data, only overlapping SNPs present in both the exposure and outcome summary datasets were retained during harmonization; missing variants without viable proxies in the outcome dataset were automatically excluded from downstream analysis to maintain data alignment.

Two-sample MR was performed using TwoSampleMR (v0.6.25). Cis-eQTLs of core paraptosis-related genes were exposures; PBC was the outcome. Weak instruments (*F* < 10) were excluded and Steiger filtering was used to assess directionality. The Wald ratio method was used for single-SNP instruments; for multi-SNP instruments (*n* ≥ 2), IVW was the primary approach with sensitivity analyses (MR-Egger intercept, MR-PRESSO, Cochran’s *Q*, leave-one-out) to assess pleiotropy, heterogeneity, and robustness.

Colocalization compared eQTLGen expression and GCST90044188 PBC signals at PDCD5 and ITPR3. A shared-variant prior of p12 = 1 × 10^−5^ and a PP.H4 ≥ 0.80 criterion were used. Additional PDCD5 MR analyses used GCST90061440 and FinnGen R12 as outcomes, with multiplicative random-effects IVW as the main estimator and MR-Egger, weighted median, weighted mode, funnel, and leave-one-out analyses for sensitivity assessment. Immune cell analyses used PDCD5 cis-eQTLs from OneK1K for CD4 NC, CD8 NC, dendritic cells, and natural killer cells. The cis-window extended ±1 Mb from the gene boundaries, with LD clumping within 10,000 kb at r^2^ < 0.01 against the 1000 Genomes European reference and exclusion of F < 10 instruments.

### 2.6. In Vivo Experimental Validation

#### 2.6.1. Materials and Reagents

Polyinosinic-polycytidylic acid (Poly I:C, Sigma-Aldrich, St. Louis, MO, USA, Cat. No. P9582); 2-Octynoic acid-bovine serum albumin (2-OA-BSA, Xi’an Qiyue Biotechnology Co., Ltd., Cat. No. Q-0009810); Complete Freund’s Adjuvant and Incomplete Freund’s Adjuvant were supplied by Shanghai Yuanye Biotechnology Co., Ltd., Shanghai, China (Cat. No. S30541 and S30542, respectively).

#### 2.6.2. Animals and Experimental Design

Twenty female C57BL/6J mice (8 weeks old; 18–22 g) were obtained from Liaoning Changsheng Biotechnology Co., Ltd. (license: SCXK(Liao) 2025-0001). After one week of acclimatization, mice were housed under controlled laboratory conditions with a 12 h light/dark cycle, regular ventilation, standard chow and water available ad libitum, and daily health monitoring. Mice were allocated by computer-generated random numbers to control and PBC model groups (*n* = 10/group). The control group received the corresponding vehicle treatment, whereas the PBC model was induced by intraperitoneal 2-OA-BSA immunization combined with Poly I:C administration [15]; complete details are in Supplementary Text S1. Blood was collected from the retro-orbital sinus under anesthesia before euthanasia; serum was separated (3000× *g*, 15 min, 4 °C) and stored at −80 °C. Detailed H&E and Masson’s trichrome protocols are in Supplementary Text S2. No animals or data were excluded; all 20 mice completed the protocol. The protocol was approved by the Animal Ethics Committee of Wuhan Hualianke Biotechnology Co., Ltd., Wuhan, China (approval no. HLK-20250102-001; approved 2 January 2025).

#### 2.6.3. Immunofluorescence (IF)

Liver tissues were fixed in 4% paraformaldehyde (PFA) at 4 °C for [24 h], followed by dehydration, paraffin embedding, and sectioning at [5 μm]. Paraffin-embedded sections were first dewaxed and rehydrated, followed by endogenous peroxidase inactivation using [3%] hydrogen peroxide. Heat-mediated antigen retrieval was performed in [1% Triton X-100 (Beyotime, Wuhan, China)/sodium citrate buffer, pH 6.0]. After permeabilization, slides were blocked with diluted normal goat serum at room temperature for 30 min. The slides were then incubated overnight ([15 h]) with the primary antibody against PDCD5 (rabbit polyclonal, ABclonal, Wuhan, China, [cat. no.A7298], dilution ratio: 1:100) in a humidified chamber at 4 °C. After rinsing with phosphate-buffered saline containing 0.05% Tween-20 (PBST), HRP-conjugated goat anti-rabbit IgG (SeraCare, Beijing, China) was applied and incubated for 1 h at 37 °C in a humidified chamber. Sections were rinsed again with PBST and incubated with AF594-conjugated tyramide working solution ([1:100] in amplification buffer) for [10 min] at room temperature, following the manufacturer’s instructions. Nuclei were counterstained with 4′,6-diamidino-2-phenylindole (DAPI, Baiqiandu, Wuhan, China). Finally, sections were mounted with antifade mounting medium. Images were captured using a fluorescence microscope (IX73, Olympus, Tokyo, Japan) with a [×20/×40] objective; PDCD5 (AF594) was excited at [561 nm] and emission was collected at [617 nm], while DAPI was excited at 405 nm. The average fluorescence intensity was quantified using ImageJ 1.54 (NIH, Bethesda, MD, USA). Three biological replicates per group were used for immunofluorescence analysis. Negative-control sections were processed identically while omitting the primary antibody.

#### 2.6.4. Western Blot (WB)

Liver homogenates were prepared in RIPA buffer with protease inhibitors. After centrifugation (12,000× *g*, 30 min, 4 °C), protein concentration was determined by BCA assay (GBCBIO, Guangzhou, China). Protein (10 µg) was resolved by SDS-PAGE and transferred to PVDF membranes, blocked with 5% skim milk (2 h), and incubated overnight at 4 °C with primary antibodies: GAPDH (Abcam, Cambridge, UK, 1:10000), PDCD5 (ABclonal, 1:2000), ALIX (ABclonal, 1:1000), CHOP (ABclonal, 1:1000), BiP/GRP78 (ABclonal, 1:1000), ERK1/2 (ABclonal, 1:2000), and phospho-ERK1/2 (Thr202/Tyr204; ABclonal, 1:1000). p-ERK1/2 was normalized to total ERK1/2; other targets were normalized to GAPDH. Membranes were incubated with HRP-conjugated secondary antibodies (Boster, Wuhan, China, 1:10000, 2 h) and visualized by enhanced chemiluminescence (Affinity). Band intensities were quantified using ImageJ 1.54. Three animals per group were used for Western blot analysis.

#### 2.6.5. Reverse Transcription Quantitative PCR (RT-qPCR)

RNA was extracted from mouse liver tissues using TRIzol^®^ Reagent (VAZYME, Nanjing, China). RNA was reverse-transcribed into cDNA, followed by quantitative PCR using the specified primers. The primer sequences used are presented in Supplementary Table S5. Supplementary Text S3 provides the detailed experimental procedure. Three animals per group were used for RT-qPCR analysis, with technical triplicates for each sample.

#### 2.6.6. Transmission Electron Microscopy (TEM)

Fresh liver tissues (approximately 1 mm^3^) were fixed in 2.5% glutaraldehyde in 0.1 M phosphate buffer (pH 7.4) for 2 h at 4 °C, followed by post-fixation in 1% osmium tetroxide for 1 h. After dehydration through a graded ethanol series (50%, 70%, 90%, and 100%) and acetone, specimens were embedded in epoxy resin. Ultrathin sections (70 nm) were cut using an ultramicrotome, stained with uranyl acetate and lead citrate, and examined using an HT7700 transmission electron microscope (Hitachi, Tokyo, Japan). Images were captured at magnifications of ×5000 to ×20,000 to assess endoplasmic reticulum morphology and mitochondrial ultrastructure. Three animals per group were examined by transmission electron microscopy.

#### 2.6.7. Serum Biochemistry and ELISA

Serum levels of alanine aminotransferase (ALT), aspartate aminotransferase (AST), alkaline phosphatase (ALP), γ-glutamyl transferase (γ-GT), total bilirubin (TBIL), direct bilirubin (DBIL), and total bile acids (TBAs) were measured using an automatic biochemical analyzer (Fully Automated Biochemical Analyzer, Shandong BIOBASE Biological Company, Model: BK280). Serum immunoglobulin A (IgA) and immunoglobulin G (IgG) concentrations were determined using commercial ELISA kits (Multi Science, catalog nos. EK274-01 for IgA and EK271-02 for IgG). Serum levels of interleukin-6 (IL-6), interleukin-1β (IL-1β), tumor necrosis factor-α (TNF-α), and interleukin-17A (IL-17A) were quantified using commercial ELISA kits (Thermo Fisher Scientific, Waltham, MA, USA; catalog nos. 88-7064-88 for IL-6, 88-7013 for IL-1β, 88-7324-88 for TNF-α, and 88-7371-88 for IL-17A) according to the manufacturers’ instructions. Serum biochemistry and ELISA analyses used samples from 10 biological animals per group.

### 2.7. HIBEpiC Culture and PDCD5 Overexpression

Human intrahepatic biliary epithelial cells (HIBEpiC; Procell, CP-H042) were cultured in HIBEpiC complete medium (CM-H042) at 37 °C with 5% CO2. Cells were transiently transfected 24 h after plating with pcDNA3.1/Neo(+)-PDCD5 or the matched empty vector. Each well of a six-well plate received 3 μg plasmid, 4.5 μL P3000, 6 μL Lipofectamine 3000, and 500 μL Opti-MEM; complete medium was replaced after 6 h. Four groups were studied: Vector, PDCD5-OE, LPS + Vector, and LPS + PDCD5-OE. LPS (Sigma-Aldrich L4391; 50 μg/mL) was applied for 12 h, starting 24 h after transfection for RT-qPCR/CCK-8 or 48 h after transfection for WB/IF. Samples were collected at 36 h or 60 h after transfection, respectively.

For CCK-8, cells were directly transfected in 96-well plates with 0.10 μg plasmid, 0.15 μL P3000, 0.20 μL Lipofectamine 3000, and approximately 16.7 μL Opti-MEM per well. Cells were incubated with CCK-8 reagent (Abmole Bioscience, Houston, TX, USA; M4839; 10 μL/well) for 2 h at 37 °C with 5% CO_2_, and absorbance was measured at 450 nm. Relative metabolic activity was normalized to the Vector control. PDCD5 mRNA was quantified by RT-qPCR and expressed as relative abundance. Three independent biological experiments per group were performed.

Cells were fixed with 4% paraformaldehyde, permeabilized with 0.1% Triton X-100, and blocked with 5% BSA for 30 min at 37 °C. Cells were incubated with the PDCD5 primary antibody for 2 h at room temperature, followed by a fluorescent secondary antibody for 30 min in the dark. Cells were counterstained with DAPI and washed three times with PBS. PDCD5 fluorescence was quantified using ImageJ, with each plotted point representing one independent biological experiment.

Cellular WB followed the lysis, BCA assay, 10 μg protein loading, electrophoresis, transfer, and immunodetection procedures described in Section 2.6.4. PDCD5, CHOP, BiP, ALIX, p-ERK, and total ERK antibodies were used under the same conditions as the animal assays. GAPDH antibody (ABclonal) and HRP-conjugated secondary antibody (Boster) were used at 1:10000. Cleaved caspase-3 was detected at approximately 17 kDa using an antibody from Abcam (EPR21032). p-ERK was normalized to total ERK, and other targets were normalized to GAPDH.

### 2.8. Statistical Methods

Data are expressed as mean ± SD. Serum biochemistry and ELISA comparisons used two-tailed Student’s *t*-tests. Animal IF, WB, and RT-qPCR comparisons used two-sided Welch *t*-tests; Holm correction was applied across five WB endpoints and separately across five RT-qPCR endpoints. The single animal IF comparison was not additionally adjusted. RT-qPCR tests were performed on log2 relative expression. Cell experiments comprised three independent, unpaired biological experiments per group. Four Welch comparisons were prespecified for each endpoint: Vector versus PDCD5-OE, Vector versus LPS + Vector, LPS + Vector versus LPS + PDCD5-OE, and PDCD5-OE versus LPS + PDCD5-OE. Holm correction was applied within each endpoint. Average OE, LPS, and interaction effects were evaluated using heteroscedastic factorial contrasts with Welch–Satterthwaite degrees of freedom, with Holm correction across the three effects per endpoint. Confidence intervals were pointwise 95% intervals. Bonferroni correction was applied to the four primary MR gene tests. Heterogeneity was assessed by Cochran’s *Q* test, horizontal pleiotropy by MR-Egger intercept and MR-PRESSO, and directionality by Steiger filtering. Animal experiments used n = 10/group for serum biochemistry and ELISA and n = 3/group for tissue assays; animal RT-qPCR used technical triplicates. Quantitative image analyses were performed using ImageJ v1.54 by blinded observers. Statistical significance was denoted as ns, *p* ≥ 0.05; * *p* < 0.05; ** *p* < 0.01; *** *p* < 0.001; and **** *p* < 0.0001, using adjusted *p* values where specified. Analyses were conducted in R v4.5.2 and GraphPad Prism v9.0.

### 2.9. Ethical Approval

The animal studies were reviewed and approved by the Animal Ethics Committee of Wuhan Hualianke Biotechnology Co., Ltd. (approval no. HLK-20250102-001; approval granted 2 January 2025). The committee oversees animal work conducted at the Wuhan Hualianke facility, where the experiments were performed on a fee-for-service basis. All procedures were conducted in accordance with the ARRIVE guidelines and the National Institutes of Health Guide for the Care and Use of Laboratory Animals. At the end of the protocol, animals received 1% sodium pentobarbital by intraperitoneal injection at a body-weight-adjusted dose to induce rapid loss of consciousness. Under deep anesthesia, euthanasia was completed by cardiectomy, ensuring rapid cessation of cardiac and respiratory activity and minimizing pain and distress. Animals were monitored daily; no specific humane endpoint criteria were predefined, and no unexpected mortality or severe adverse events occurred. Only female C57BL/6J mice were used because PBC predominantly affects females; this rationale and the limitation of single-sex modeling are discussed in the Section 4.

## 3. Results

### 3.1. Screening of Key Genes

Differential expression analysis identified 1423 DEGs (Table S6) between PBC and healthy control samples (Figure 1A,B). Intersection of these DEGs with 66 curated paraptosis-related genes yielded 8 candidate genes (Figure 1C). To identify the core candidate genes, we developed an integrated machine learning framework comprising 113 model combinations. As illustrated in the model performance heatmap (Figure 1D), the LASSO + RF ensemble model demonstrated the highest AUC ranking across the training and three external evaluation cohorts. SHAP analysis was subsequently applied to the LASSO + RF model to assess feature importance. The SHAP summary bar plot (Figure 1E) and beeswarm plot (Figure 1F) visualized the relative importance and directional impact of these four genes, illustrating their predictive contributions. Expression profiling confirmed significant dysregulation of these four core genes in PBC samples (Figure 1G). Furthermore, ROC curve analysis evaluated their apparent individual discriminative performance for PBC, yielding AUCs of 0.832 (AKT1), 0.680 (PEBP1), 0.714 (PDCD5), and 0.653 (ITPR3), respectively (Figure 1H). The fitted-model comparison included 45 retained combinations. The LASSO + RF AUCs were 0.990 in GSE119600, 0.722 in GSE159676, 0.725 in GSE61260, and 0.667 in GSE93170. Internal validation yielded an out-of-fold AUC of 0.781 (95% CI 0.703–0.859), a Brier score of 0.178, and a calibration slope of 0.75. Sensitivity and specificity were 0.778 and 0.660 at the Youden threshold of 0.594 (Figure S6).

MR analysis linked genetically predicted PDCD5 (OR = 0.53, 95% CI 0.35–0.81; Bonferroni *p* = 0.014) and ITPR3 expression (OR = 0.70, 95% CI 0.55–0.88; Bonferroni *p* = 0.011) with lower PBC odds (Figure 1I). The retained instrument counts were 12 for AKT1, 20 for PEBP1, 38 for PDCD5, and 57 for ITPR3; gene-specific minimum F statistics ranged from 30.1 to 31.5 (Tables S3 and S4). When selecting the target for downstream experiments, we considered genetic association, SHAP contribution, and expression consistency. The inverse PDCD5 MR association was consistent with its lower expression in PBC. PDCD5 was therefore prioritized for subsequent animal and cellular experiments.

Colocalization yielded PP.H3 = 0.001 and PP.H4 = 0.999 for PDCD5, compared with PP.H3 = 0.488 and PP.H4 = 0.218 for ITPR3 (Figure S1). Thus, PDCD5 met the PP.H4 ≥ 0.80 criterion, whereas ITPR3 did not. In GCST90061440, the 16-SNP IVW estimate for PDCD5 was OR 0.827 (95% CI 0.731–0.935; *p* = 0.00245); Cochran’s *Q* and MR-Egger intercept *p* values were 0.375 and 0.792, respectively. FinnGen R12 yielded OR 0.82 (95% CI 0.71–0.95; *p* = 0.00787; Figures S2 and S3). Immune cell IVW estimates were OR 0.628 (95% CI 0.412–0.957; *p* = 0.0304; 28 SNPs) for CD4 NC, 0.446 (0.201–0.990; *p* = 0.0473; 8 SNPs) for CD8 NC, 0.219 (0.071–0.673; *p* = 0.00800; 5 SNPs) for dendritic cells and 0.403 (0.163–0.994; *p* = 0.0484; 8 SNPs) for natural killer cells (Figures S4 and S5). Weighted-median and weighted-mode estimates were not significant. These nominal associations were directionally consistent with the whole-blood estimate.

### 3.2. PBC Model Establishment and Validation

The 2-OA-BSA/Poly I:C model combines xenobiotic-induced loss of tolerance with innate immune stimulation and reproduces selected serological and histological features of autoimmune cholangitis [15]. To confirm the successful establishment of the PBC model, serum biochemical markers, immunoglobulins, inflammatory cytokines, and liver histopathology were evaluated. Compared with control mice, PBC model mice exhibited significantly elevated serum levels of ALP, γ-GT, TBAs, TBIL, and DBIL (all *p* < 0.001; Figure 2A), indicative of cholestatic liver injury. Serum IgA and IgG concentrations were markedly increased in the model group (*p* < 0.001; Figure 2B), consistent with the autoimmune humoral response characteristic of PBC. Pro-inflammatory cytokines, including IL-6, IL-1β, TNF-α, and IL-17A, were also significantly elevated (all *p* < 0.001; Figure 2C). H&E staining revealed prominent portal inflammation with lymphocytic infiltration and bile duct epithelial injury in model mice, and Masson’s trichrome staining confirmed increased periportal collagen deposition compared with controls (Figure 2D).

### 3.3. Ultrastructural Alterations and PDCD5 Expression in the Liver

TEM revealed pronounced ultrastructural abnormalities in hepatocytes of PBC model mice. Compared with the control group, hepatocytes from model mice displayed marked dilation of the endoplasmic reticulum, as well as enlarged mitochondria with disrupted cristae (Figure 3A). These morphological features overlap with those reported in paraptosis but are not specific to this form of cell death. Immunofluorescence staining of PDCD5 showed a substantially reduced fluorescence signal in liver tissues of PBC model mice relative to controls (Figure 3B), and quantitative analysis of mean fluorescence intensity confirmed a significant decrease in PDCD5 expression (*p* < 0.001; Figure 3C).

### 3.4. Paraptosis-Related Protein and mRNA Expression in the Liver

To further characterize paraptosis-associated molecular changes, Western blot and RT-qPCR analyses were performed on liver tissues. Western blot analysis confirmed that PDCD5 and ALIX protein levels were significantly downregulated in the PBC model group (*p* < 0.001; Figure 4A,B). Concurrently, the ER stress markers CHOP and BiP were markedly upregulated (both *p* < 0.001), and the ratio of phosphorylated ERK1/2 to total ERK1/2 was significantly increased (*p* < 0.001), indicating increased ERK phosphorylation. At the transcriptional level, RT-qPCR revealed significantly reduced mRNA expression of PDCD5 and ALIX, accompanied by elevated CHOP and BiP mRNA in the model group (Holm-adjusted *p* < 0.01; Figure 4C). ERK mRNA did not differ significantly (adjusted *p* = 0.400).

### 3.5. PDCD5 Overexpression Attenuates LPS-Associated Changes in HIBEpiC

The experimental design and treatment schedule are summarized in Figure 5A. PDCD5 overexpression increased PDCD5 mRNA, fluorescence intensity, and protein abundance (Figure 5B,D–G). Under LPS exposure, relative metabolic activity measured by CCK-8 was higher in the PDCD5-OE group than in the Vector group (0.69 ± 0.04 versus 0.46 ± 0.04; Holm-adjusted *p* = 0.00429; Figure 5C). The interaction between LPS treatment and PDCD5 overexpression was significant (*p* = 0.00149). Under LPS exposure, PDCD5 overexpression reduced CHOP and BiP protein levels and the p-ERK/ERK ratio and increased ALIX protein levels (adjusted *p* = 0.000264, 0.00462, 0.0363 and 0.0000975, respectively; Figure 5H–K). Interactions were significant for CHOP, BiP, and ALIX, but not for p-ERK/ERK (*p* = 0.0503). Cleaved caspase-3 did not differ significantly in the four prespecified comparisons (all adjusted *p* = 1.00; Figure 5L). Together, these findings show attenuation of LPS-associated metabolic and stress-related changes after PDCD5 overexpression.

## 4. Discussion

This study provides multi-omics and experimental evidence implicating PDCD5 as a candidate associated with PBC. We combined transcriptomic profiling, machine learning, and MR to prioritize genes for experimental investigation. No single omics layer can reliably distinguish causal drivers from downstream responses in complex diseases [16]. Beginning with differential expression analysis of 66 paraptosis-related genes, we used an integrated machine learning framework comprising 113 algorithm combinations to rank candidates by discriminative performance. The LASSO + RF ensemble emerged as the optimal classifier, consistent with growing evidence that ensemble models combining regularization (LASSO) with decision tree-based methods (RF) achieve superior robustness in high-dimensional biomedical feature selection [17,18]. SHAP analysis further decomposed model predictions into feature-level contributions, providing interpretable importance rankings that guided downstream target prioritization, an approach increasingly endorsed for biomarker discovery in liver diseases [18,19]. Transcriptomic dysregulation may reflect either causal drivers or compensatory responses to the disease state; ML-derived feature importance alone cannot resolve this directionality. We therefore applied two-sample MR to evaluate genetically predicted expression of the four ML-prioritized core genes. This two-stage strategy, ML for candidate nomination followed by MR for genetic prioritization, integrates complementary evidence beyond single-modality approaches [16,20]. Among the four candidates, only the MR associations for PDCD5 and ITPR3 remained significant after Bonferroni correction. PDCD5 was prioritized for downstream experiments based on its inverse MR association, SHAP contribution, and concordant lower expression in PBC.

PDCD5 (Programmed Cell Death 5) is an evolutionarily conserved protein originally identified as TFAR19 for its pro-apoptotic function via mitochondrial translocation. Subsequent work has considerably expanded its known functional repertoire: PDCD5 interacts with the Tip60-p53 axis, modulates NF-kappaB signaling, and regulates both apoptosis and autophagy [21]. Pdcd5 knockout in mice causes embryonic lethality with severe placental defects, indicating an essential developmental role [22]. More recently, PDCD5 has been shown to promote pyroptosis in hepatocellular carcinoma by binding Smad2/3 and inhibiting TGF-beta signaling [23], a pathway highly relevant to PBC-associated fibrosis. Collectively, these findings suggest that PDCD5 functions as a context-dependent regulator of multiple cell death and survival pathways, pro-death in neoplastic settings but potentially homeostatic or protective in certain non-malignant contexts. Our findings support this context-dependent role by linking reduced hepatic PDCD5 expression to stress-related changes in PBC and showing attenuation of LPS-associated injury after PDCD5 overexpression in HIBEpiC. Previous work showed that PDCD5 overexpression enhanced TAJ/TROY-induced paraptosis-like death in 293T cells [24]. Differences in cellular and stimulus context may contribute to the contrasting observations, but this possibility was not tested here.

ITPR3 (Type 3 Inositol 1,4,5-Trisphosphate Receptor), the second gene with an MR association that remained significant after Bonferroni correction, provides an additional candidate for investigation in PBC. ITPR3 is the principal ER Ca2^+^ release channel in cholangiocytes and localizes to ER–mitochondria contact sites, where it mediates mitochondrial Ca2^+^ uptake [25]. Intracellular Ca2^+^ dyshomeostasis, particularly mitochondrial Ca2^+^ overload, is a well-established trigger of paraptosis, and may link ER and mitochondrial stress [26]. However, ITPR3 was not directly evaluated by targeted qPCR or Western blot in this study, and its co-identification does not establish a shared mechanism with PDCD5. AKT1 had the largest mean absolute SHAP contribution and an apparent single-gene AUC of 0.832, whereas its MR association was not significant. AKT-dependent biliary secretory responses provide biological context [27]; its role in PBC requires direct investigation.

The in vivo findings identify morphological and molecular changes associated with cellular stress. TEM revealed ER and mitochondrial abnormalities, including ER dilation and mitochondrial swelling with disrupted cristae, mirroring the original descriptions by Sperandio et al. [6]. These features were accompanied by reduced expression of ALIX, a regulator implicated in experimental paraptosis [7], increased levels of the ER stress markers CHOP and BiP, and increased ERK phosphorylation. The concurrent dysregulation of PDCD5 and ALIX alongside MAPK pathway activation overlaps with features described in paraptosis models [6,7]. Although paraptosis research has largely centered on oncology, associated features such as ER dilation, mitochondrial swelling, and MAPK/ERK signaling closely mirror cellular stress features of cholestatic liver injury [8].

The HIBEpiC experiments extend the tissue observations by directly manipulating PDCD5 in a disease-relevant epithelial cell type. Increased metabolic activity and lower CHOP/BiP expression after PDCD5 overexpression suggest attenuation of LPS-associated stress. The accompanying restoration of ALIX and reduction in p-ERK/ERK connect this functional response to the molecular changes observed in liver tissue. However, the nonsignificant p-ERK/ERK interaction and absence of pathway inhibition leave ERK dependence unresolved.

Our findings contribute to research on PBC biomarkers and therapeutic targets. Recent proteome-wide MR studies in PBC have identified several plasma proteins with causal associations, yet few have been validated in vivo [20]. Colocalization supports a shared PDCD5 expression–PBC signal, and the additional GWAS and immune-cell MR estimates show directionally consistent associations (Figures S1–S5). These analyses complement the whole-blood result but do not identify a cholangiocyte-specific mechanism. However, the intracellular nature of PDCD5 and its lack of a canonical small-molecule-binding domain present challenges for direct pharmacological targeting that remain to be addressed.

Several limitations warrant consideration. The PBC GWAS cohorts were of European ancestry, whereas eQTLGen was predominantly European; generalizability to East Asian cohorts requires confirmation. eQTL instruments were derived from peripheral blood rather than liver tissue, and tissue-specific eQTL effects may exist. The PBC mouse model, while recapitulating key serological and histological features, was limited to female mice, consistent with the marked female predominance of PBC but precluding assessment of sex-specific effects. The current paraptosis gene set (*n* = 66) may not comprehensively capture all paraptosis regulators, as the molecular boundaries of this cell death modality continue to be refined [8]. The external evaluation cohorts contributed to joint preprocessing and model selection, and the initial gene screen preceded internal validation; residual optimism remains possible. Cross-tissue differences in cell composition may contribute to prediction, and cohort-level ComBat adjustment cannot distinguish these biological differences from technical batch effects. The animal observations derive from whole-liver or hepatocyte TEM and do not establish cholangiocyte-specific PDCD5 localization. Although the HIBEpiC experiments directly manipulated PDCD5, CCK-8 measures metabolic activity, and the absence of a cleaved caspase-3 difference does not exclude other death pathways. Knockdown, rescue, MEK/ERK inhibition, and cell-level vacuolation or TEM were not performed. These limitations leave the death pathway specificity and in vivo cellular mechanism unresolved.

## 5. Conclusions

These findings integrate computational prediction, genetic association, and experimental evidence to nominate PDCD5 as a candidate associated with PBC. PDCD5 overexpression attenuated LPS-associated loss of metabolic activity and stress-related molecular changes in cultured human biliary epithelial cells. Future studies should examine its death pathway specificity and ERK dependence in cholangiocyte-specific models and evaluate its relevance in independent PBC cohorts.

## Figures and Tables

**Figure 1:**
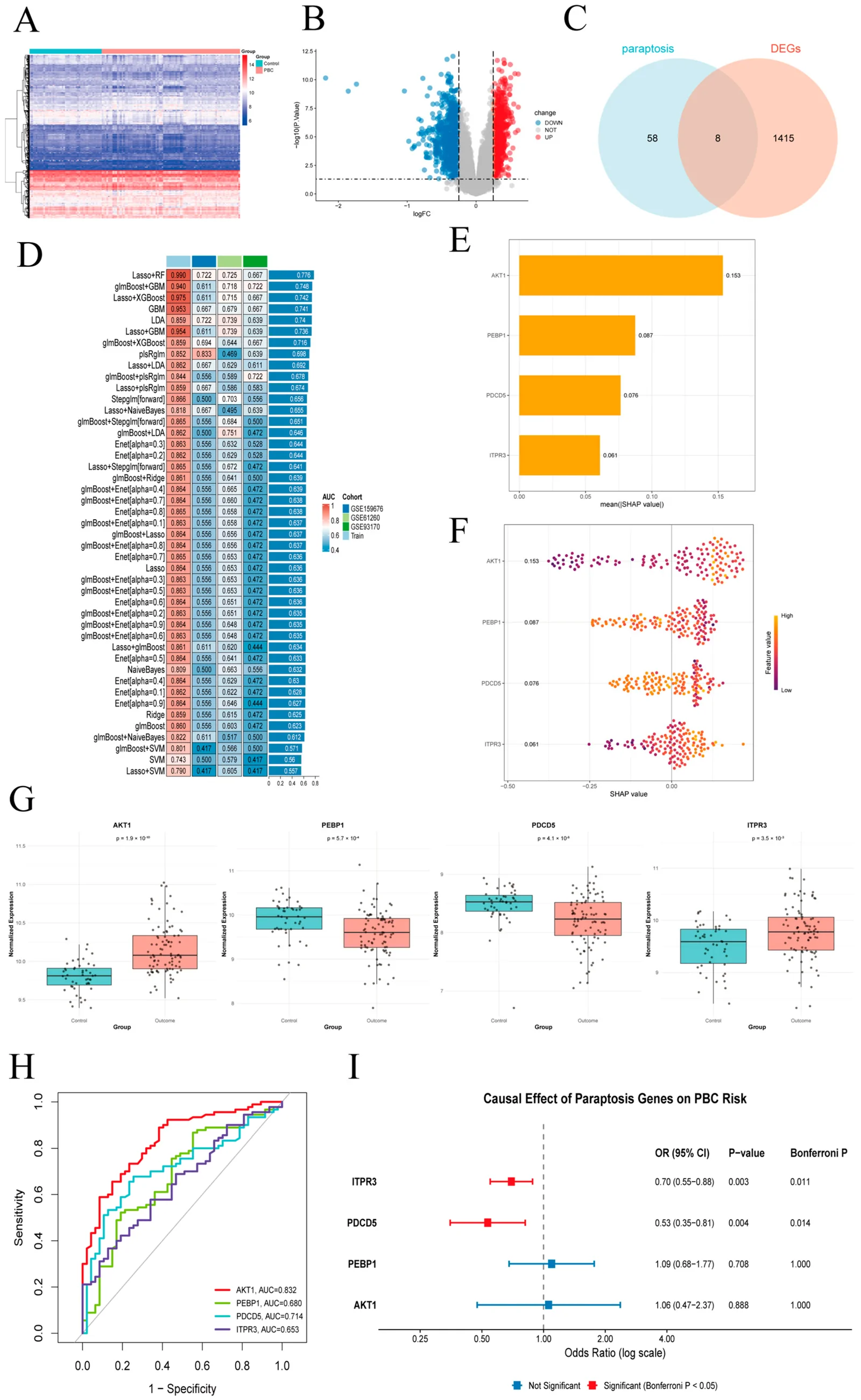
Multi-omics pipeline integrating machine learning and Mendelian randomization prioritizes paraptosis-related genes in PBC. (**A**) Heatmap and (**B**) volcano plot showing DEGs in the GSE119600 dataset. (**C**) Venn diagram intersecting DEGs with paraptosis-related genes. (**D**) Heatmap of the AUC values for retained machine learning model combinations across the training and external evaluation cohorts. (**E**) SHAP feature importance bar plot and (**F**) SHAP beeswarm plot. (**G**) Boxplots showing the normalized expression of the core genes in PBC vs. control samples. (**H**) ROC curves showing the apparent discriminatory performance of the core genes in the GSE119600 dataset. (**I**) Forest plot of two-sample MR estimates for the core genes; PDCD5 and ITPR3 were associated with lower PBC odds (Bonferroni *p* < 0.05). In (**F**), each point represents a sample-specific SHAP value; separated AKT1 clusters may reflect nonlinear tree thresholds and feature interactions rather than bimodal expression.

**Figure 2:**
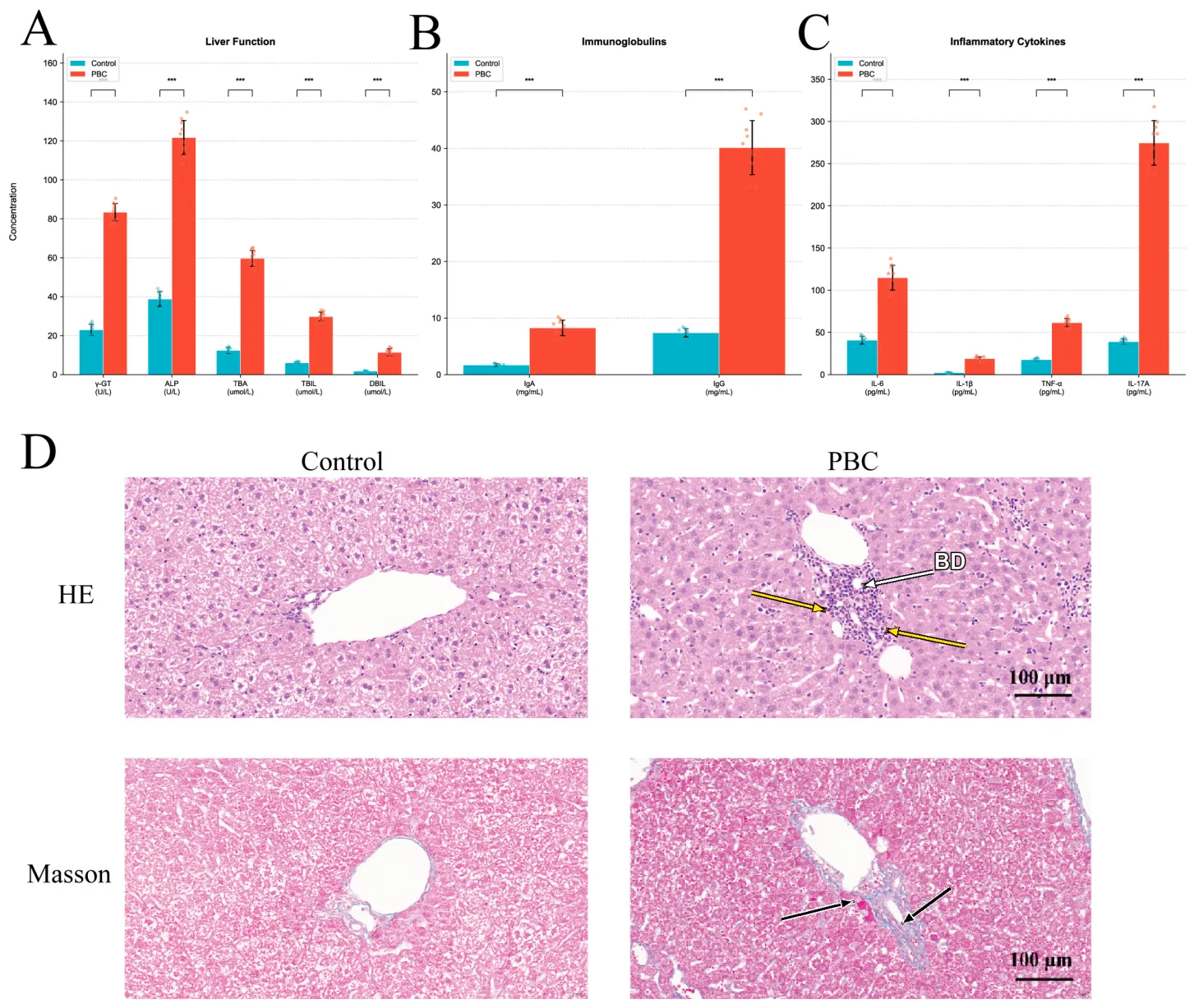
Evaluation of liver function, inflammatory responses, and pathological changes in a mouse model of PBC. (**A**) Serum levels of liver function markers, including γ-GT, ALP, TBAs, TBIL, and DBIL, in control and PBC model mice. (**B**) Serum concentrations of immunoglobulins (IgA and IgG). (**C**) Serum levels of inflammatory cytokines (IL-6, IL-1β, TNF-α, and IL-17A). (**D**) Hematoxylin–eosin (HE) and Masson’s trichrome staining of liver sections from control and PBC model mice (scale bar: 100 μm). Data are presented as mean ± SD (*n* = 10 for panels (**A**–**C**); *n* = 3 for panel (**D**)). *** *p* < 0.001 by two-tailed Student’s *t*-test. In (**D**), BD denotes a bile duct; yellow arrows indicate the inflammatory infiltrate in the PBC H&E panel, and black arrows indicate blue-stained collagen in the PBC Masson panel.

**Figure 3:**
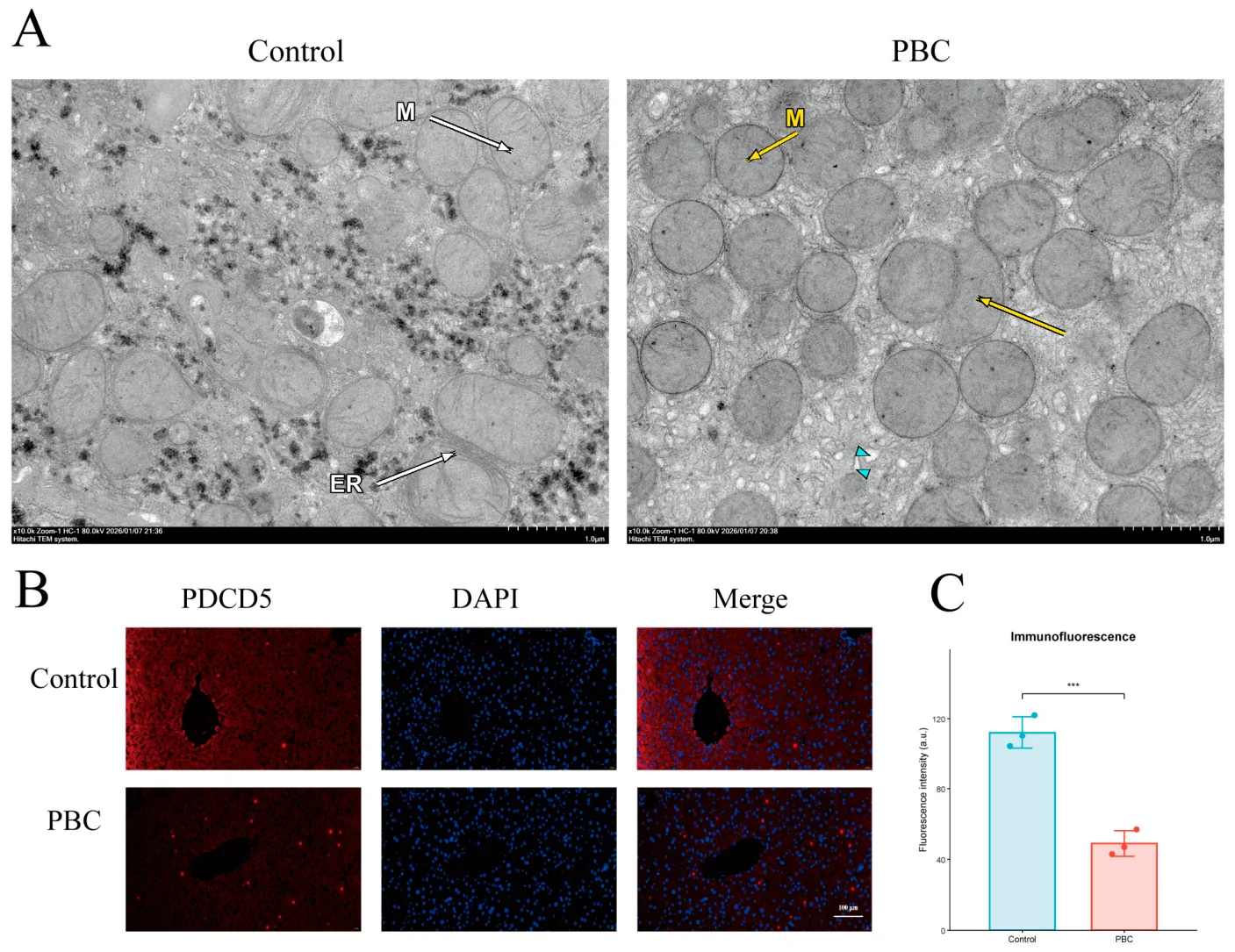
Ultrastructural alterations and PDCD5 expression in liver tissues of PBC model mice. (**A**) Representative transmission electron microscopy (TEM) images showing ultrastructural changes of organelles in liver tissues from control and PBC model mice. (**B**) Immunofluorescence staining of PDCD5 (red) and DAPI counterstaining (blue) showing PDCD5-associated fluorescence in liver tissues (scale bar: 100 μm). (**C**) Quantitative analysis of PDCD5 fluorescence intensity in control and model mice. Data are presented as mean ± SD (*n* = 3). *** *p* < 0.001 by two-tailed Welch *t*-test. The individual points represent three animals per group. In (**A**), M denotes mitochondria and ER denotes endoplasmic reticulum. Yellow arrows indicate mitochondrial profiles with reduced cristae definition, and cyan arrowheads indicate dilated cisternal profiles consistent with ER dilation in the PBC panel.

**Figure 4:**
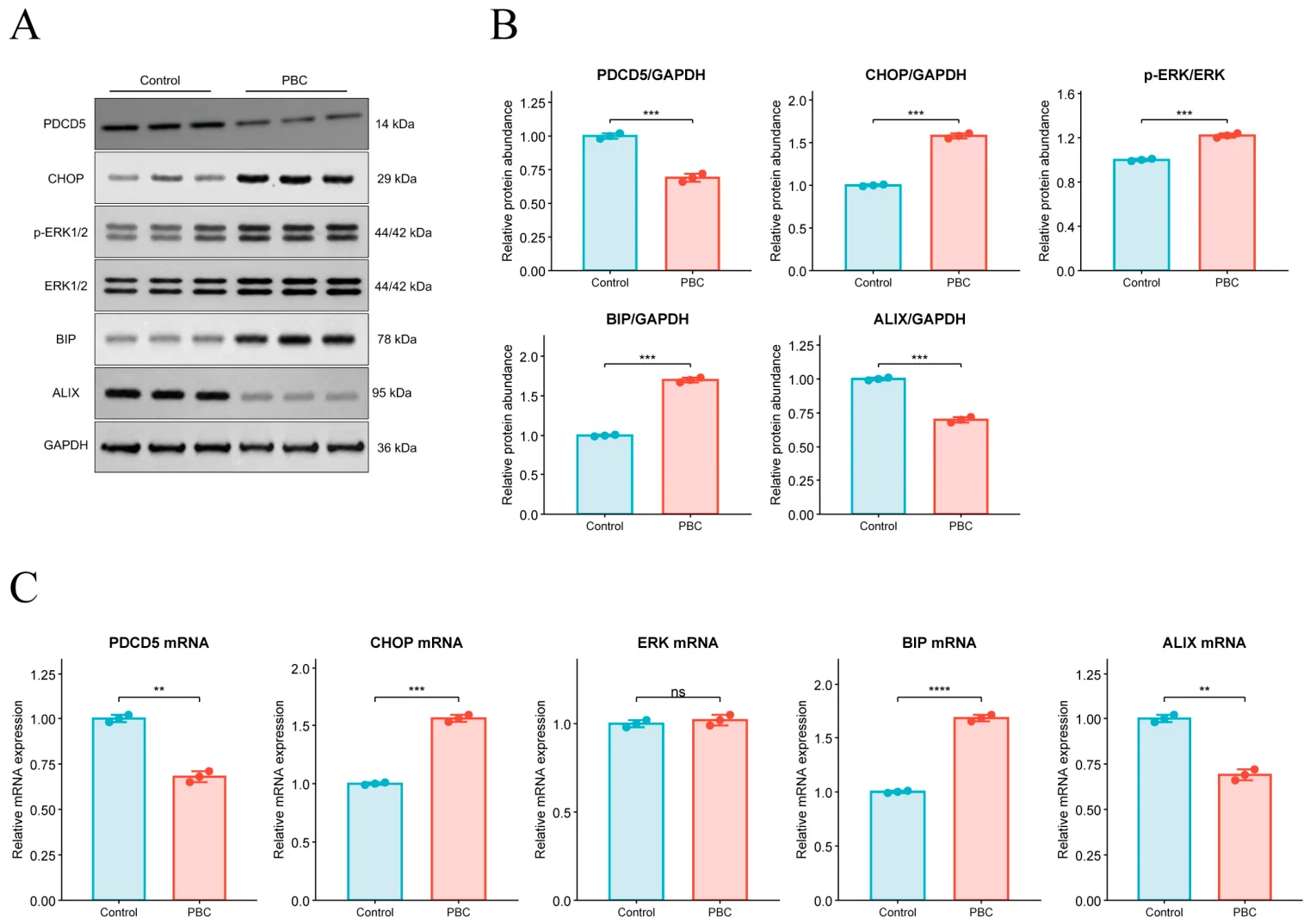
Expression levels of PDCD5, ALIX, and ER stress-related markers in the liver of PBC model mice. (**A**) Western blot analysis of PDCD5, CHOP, p-ERK1/2, ERK1/2, BiP, and ALIX protein expression in liver tissues from control and PBC model mice, with GAPDH as a loading control. (**B**) Quantitative analysis of PDCD5, CHOP, BiP, and ALIX protein levels relative to GAPDH, and the ratio of p-ERK1/2 to total ERK1/2. (**C**) Quantitative RT-PCR analysis of mRNA expression levels for PDCD5, CHOP, ERK1/2, BiP, and ALIX in liver tissues. Data are presented as mean ± SD (*n* = 3). Two-sided Welch tests were Holm-adjusted across five protein endpoints and separately across five transcript endpoints; RT-qPCR tests used log2 expression. ns, adjusted *p* ≥ 0.05; ** *p* < 0.01; *** *p* < 0.001; **** *p* < 0.0001. Individual observations are shown.

**Figure 5:**
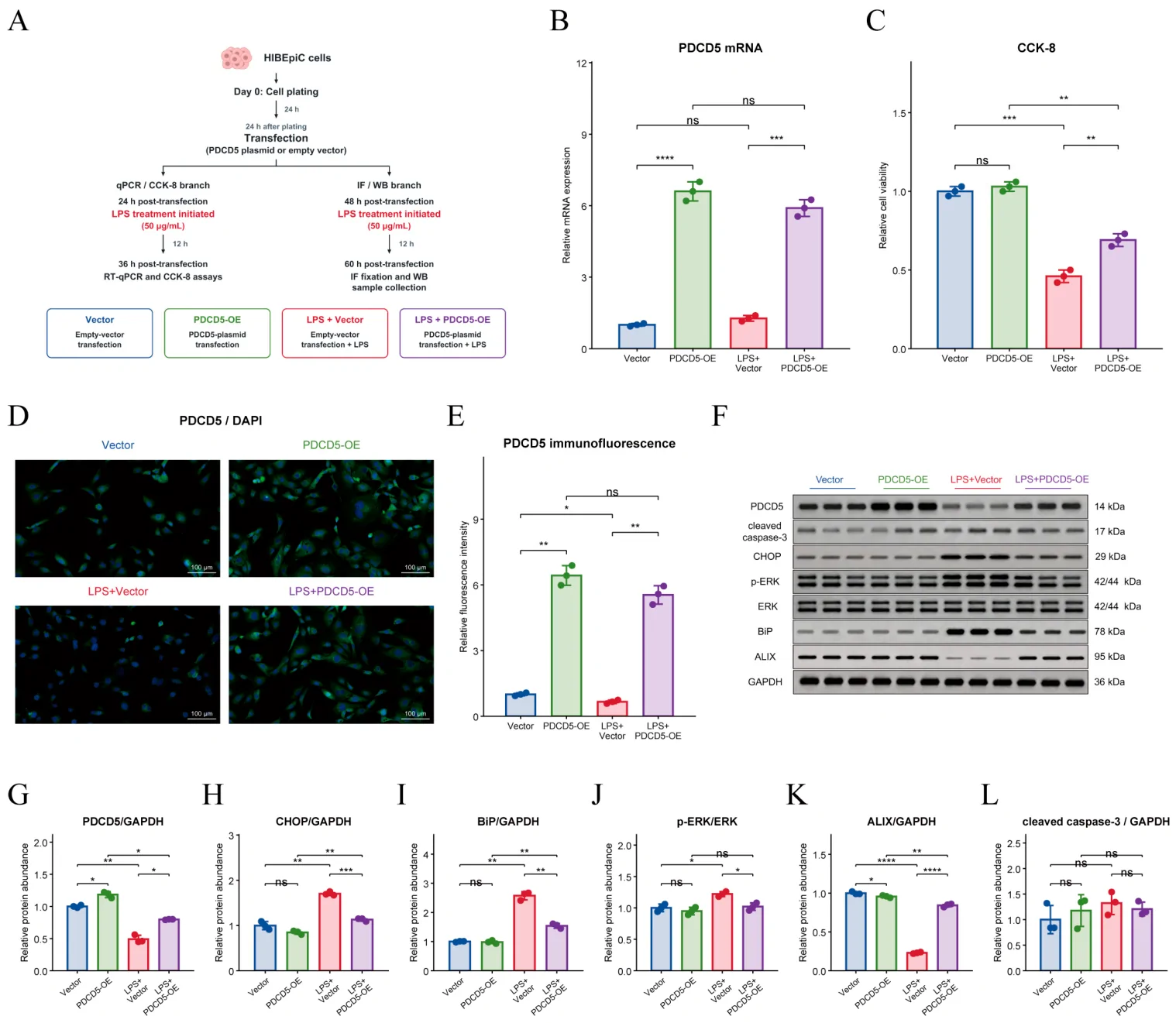
Effects of PDCD5 overexpression in LPS-treated HIBEpiC. (**A**) Experimental timeline, with RT-qPCR/CCK-8 at 36 h and IF/WB at 60 h after transfection. (**B**) PDCD5 mRNA. (**C**) Relative CCK-8 metabolic activity. (**D**) PDCD5 immunofluorescence (green) and DAPI (blue); scale bar, 100 μm. (**E**) PDCD5 fluorescence intensity. (**F**) Western blots, with three lanes per group. (**G**–**L**) PDCD5/GAPDH, CHOP/GAPDH, BiP/GAPDH, p-ERK/ERK, ALIX/GAPDH, and cleaved caspase-3/GAPDH. Data are mean ± SD with individual points (three independent biological experiments per group). Four prespecified two-sided Welch comparisons were Holm-adjusted within each endpoint; RT-qPCR tests used log2 expression. ns, adjusted *p* ≥ 0.05; * *p* < 0.05; ** *p* < 0.01; *** *p* < 0.001; and **** *p* < 0.0001.

**Table 1:** Public datasets and reference resources used in this study.

Resource and Role	Tissue and Platform	Sample Size and Population	Source Preprocessing and Batch Handling
GSE119600	Whole blood; Illumina HT-12 v4 (GPL10558)	90 PBC/47 controls; Polish Caucasians	lumi quantile normalization; no background correction
GSE93170	CD4+ T cells; Agilent 8 × 60 K (GPL14550)	6 PBC/6 controls; ancestry NR	GeneSpring 90th-percentile normalization
GSE159676	Liver; Affymetrix Gene 1.0 ST (GPL6244)	3 PBC/6 references; ancestry NR	Processed GEO matrix; normalization algorithm NR
GSE61260	Liver; Affymetrix Gene 1.1 ST (GPL11532)	11 PBC/38 controls; German Caucasians	oligo RMA; log2 expression
eQTLGen Phase I	Blood/PBMCs; expression arrays and RNA-seq	Up to 31,684; predominantly European	Cohort-specific normalization and covariate adjustment; meta-analysis
GCST90044188	UK Biobank; genotyping arrays/imputation	121 cases/456,227 controls; European	Genotype QC/imputation; fastGWA-GLMM
GCST90061440	Five GWAS panels; genotyping arrays/imputation	8021 cases/16,489 controls; European	QC; Eagle2 phasing/1000 Genomes imputation; fixed-effect meta-analysis/genomic control
FinnGen R12 CHIRBIL_PRIM	Registry-linked genotypes; arrays/imputation	760 cases/372,273 controls; Finnish	QC/imputation; REGENIE adjusted for age, sex, 10 PCs, chip and legacy batch
OneK1K (GSE196830)	PBMCs; 10x v2/NovaSeq; Global Screening Array	982 donors; ~1.27 million cells; European	Cell Ranger/demuxlet; SCTransform with pool/technical covariates; cell-type-specific eQTL mapping
1000 Genomes Phase 3	Sequencing and array genotypes	503 European individuals	Phased GRCh37 reference for LD clumping

GEO counts indicate analyzed subsets (Table S2); reference tissue definitions are given in Section 2.2. Population labels follow source reports. NR, not reported; additional technical batch correction was not specified in the four GEO records. Study-level ComBat adjustment and standardization are described in Section 2.3. PBMC, peripheral blood mononuclear cell; RMA, robust multi-array average; QC, quality control; PC, principal component; LD, linkage disequilibrium. Resource names link to source records.

## Data Availability

The datasets analyzed during the current study are available in the following public repositories: cis-eQTL data from the eQTLGen database: https://www.eqtlgen.org/cis-eqtls.html (accessed on 10 May 2026); GWAS summary statistics for PBC were downloaded from the GWAS Catalog (accession ID: GCST90044188); transcriptomic datasets were retrieved from the Gene Expression Omnibus (GEO) under accession numbers GSE119600, GSE159676, GSE61260, and GSE93170. All these datasets are publicly accessible without specific access permissions. The experimental data generated from mouse models and HIBEpiC in this research are available from the corresponding author upon reasonable request. Supplementary analyses also use GCST90061440, FinnGen R12, and OneK1K immune cell eQTL data.

## Supplementary Materials

The following supporting information can be downloaded at: https://www.mdpi.com/article/10.3390/life16101583/s1, Table S1: List of paraptosis-related genes; Table S2: Mouse RT-qPCR primer sequences; Table S3: Differential expression results; Table S4: Transcriptomic sample identifiers and group labels; Table S5: Genetic instruments used in the primary MR analyses; Table S6: Primary MR estimates and sensitivity analyses; Texts S1–S3: Detailed protocols for animal modeling, histological analysis, and RT-qPCR; Figure S1: Colocalization analyses; Figures S2 and S3: PDCD5 MR analyses using additional PBC GWAS datasets and corresponding sensitivity analyses; Figures S4 and S5: Immune-cell-specific PDCD5 MR analyses and corresponding sensitivity analyses; Figure S6: Internal validation of the machine learning model. Computational code, sample labels, and model predictions are provided as supplementary files.
