# Supplementary material for: Integrated Multi-Omics and Experimental Evidence Implicates PDCD5 as a Candidate Gene Associated with Primary Biliary Cholangitis
Source: Life (Basel). 2026 Sep 22;16(10):1583. doi: 10.3390/life16101583 (PMC13705998; doi:10.3390/life16101583)
Supplement: Supplementary file 1 [file life-16-01583-s001.zip › life-4458404_Supplementary.pdf]

life

MDPI

Supplementary Materials

Text S1: PBC murine model establishment

Under sterile experimental conditions, mice were first immunized with a 4 g/L 2-OA-BSA conjugate mixed in 10 g/L Freund's Complete Adjuvant (containing Mycobacterium tuberculosis H37Ra). Subsequent booster immunizations were performed every 2 weeks using 2-OA-BSA combined with Freund's Incomplete Adjuvant. After the initial immunization, PolyI:C was dissolved in phosphate-buffered saline (PBS) and administered via intraperitoneal injection at a dose of 5 mg/kg, once every three days, for a total of 12 weeks. All medicinal solutions were rewarmed to 37 °C prior to intraperitoneal injection. Control animals received the corresponding vehicle treatment on the same schedule. Mice were checked daily throughout the protocol.

Text S2: Histopathological analysis

The liver of mice was stained with hematoxylin and eosin (H&E) and Masson's trichrome reagents. Subsequently, their tissue morphology was examined using a standard optical microscope.

H&E: Tissues that had previously been fixed for 48 hours were first dehydrated using a gradient alcohol dehydration method (gradient alcohol dehydration method). The tissue block is then mixed with anhydrous ethanol with xylene to make the tissue block transparent. The transparent tissue blocks were soaked in wax (1h each) by 3 cylinders of paraffin (60 °C), and the above 3 experimental steps were completed in the biological tissue dehydrator. After dehydration, wax-soaked tissue blocks were wrapped in paraffin blocks. The embedded blocks were sectioned using a microtome, and the cut tissue sections were spread in a water bath at 40°C, then attached to the slides and baked in the 60°C oven for 3 hours. The paraffin sections are then deparaffinized. The sections were stained with Mayer's hematoxylin staining solution and 1% water-soluble eosin staining solution.

Masson: Prepared paraffin sections were sequentially deparaffinized in xylene Ⅰ and Ⅱ for 10 min each, rehydrated in a graded ethanol series (100%, 95%, 85%, 75%) for 5 min per concentration, and rinsed twice with distilled water (3 min each time). 50 μl hematoxylin staining solution was added dropwise to each section, stained for 5 min, then the solution was discarded and the sections were washed with distilled water for 5 min. The sections were differentiated with an appropriate amount of acid differentiation solution for 30 s, rinsed with running tap water for bluing for 10 min, and quickly rinsed with distilled water for a few seconds. 50 μl ponceau-fuchsin staining solution was added dropwise, stained for 10 min, the solution was discarded, and the sections were rinsed with distilled

water for a few seconds (≤10 s), then differentiated with phosphomolybdic acid differentiation solution for 2 min and the solution was discarded. 50 μl fast green staining solution was added dropwise, stained for 1 min, the solution was discarded, the sections were rinsed with distilled water for a few seconds, then differentiated with acid differentiation solution for 1 min and the solution was discarded. The sections were quickly dehydrated in 70%, 80%, 90% ethanol and absolute ethanol for 10 s each, cleared in xylene three times (1~2 min each time), mounted with neutral balsam, and observed under a microscope.

Text S3: RNA extraction and Real-Time quantitative PCR

Samples (n = 3 biological replicates for each treatment) were collected from each treatment to extract total RNA using TRIzol reagent (VAZYME, Nanjing, China). RNA quality was assessed using 1% agarose electrophoresis and by measuring the 260/280 nm absorbance ratios. After purification using 4 × gDNA wiper Mix, to remove genomic DNA contamination, the total RNA was reverse-transcribed into cDNA and the gene transcription levels were analyzed using a SYBR Green Master Mix kit (VAZYME, Nanjing, China). The program was 95 °C for 10 min, followed by 40 cycles at 95 °C for 15 s, 60 °C for 60 s, and 95 °C for 15 s. The gene primer sequences were designed on the online Primer5 software. The mRNA expression levels of PDCD5, ALIX, CHOP, ERK1/2, and BiP were normalised to GAPDH and calculated using the $2-\Delta\Delta Ct$ method.

Supplementary Tables

Table S1. List of 66 paraptosis-related genes.

Table S2. Mouse RT-qPCR primer sequences.

Table S3. Differential-expression results.

Table S4. Exact transcriptomic sample identifiers and group labels.

Table S5. Primary MR SNP instruments, alleles, effects, sample-size fields, F statistics and variance explained.

Table S6. Primary MR estimates and sensitivity results.

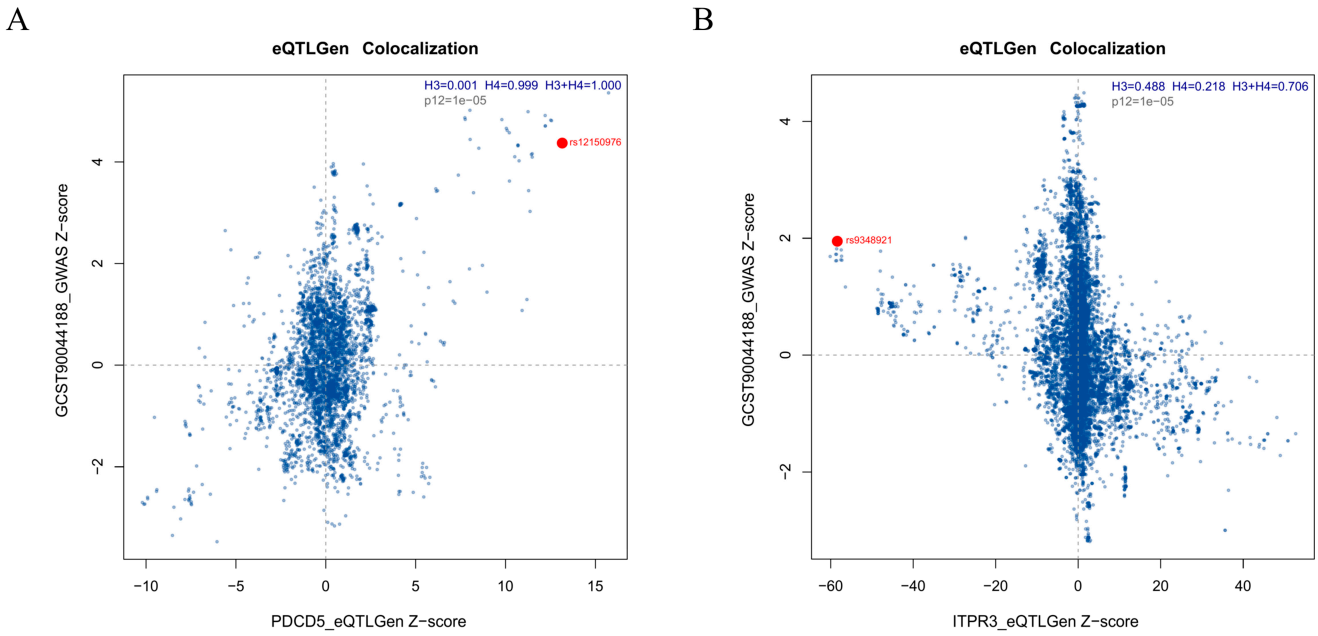

Figure S1. Colocalization of eQTLGen expression and GCST90044188 PBC signals. (A) PDCD5; (B) ITPR3. With p12 = 1×10^{-5}, PP.H3/PP.H4 are 0.001/0.999 for PDCD5 and 0.488/0.218 for ITPR3.

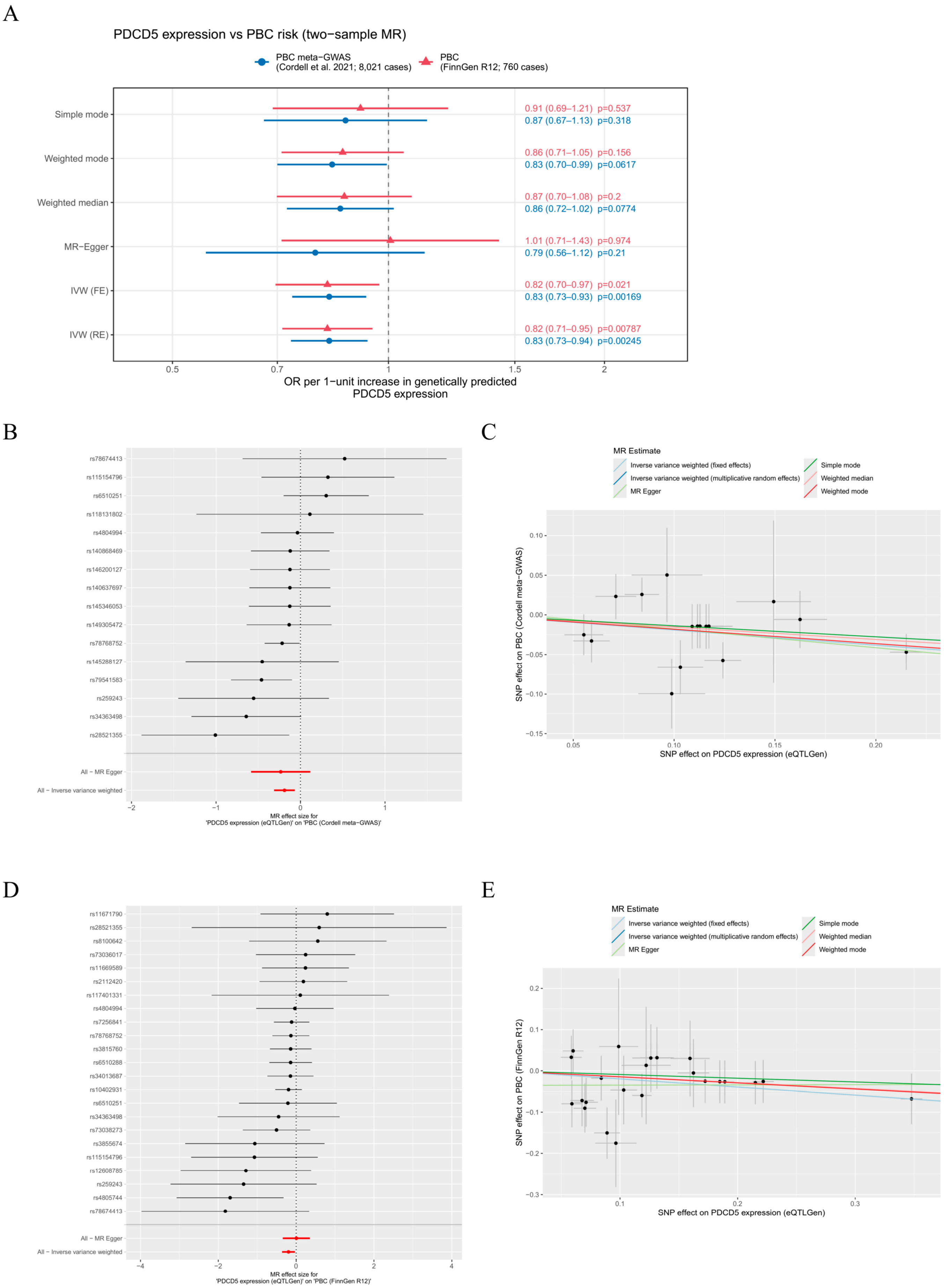
A
PDCD5 expression vs PBC risk (two−sample MR)
PBC meta−GWAS (Cordell et al. 2021; 8,021 cases)
PBC (FinnGen R12; 760 cases)
Simple mode
Weighted mode
Weighted median
MR−Egger
IVW (FE)
IVW (RE)
0.91 (0.69–1.21) p=0.537
0.87 (0.67–1.13) p=0.318
0.86 (0.71–1.05) p=0.156
0.83 (0.70–0.99) p=0.0617
0.87 (0.70–1.08) p=0.2
0.86 (0.72–1.02) p=0.0774
1.01 (0.71–1.43) p=0.974
0.79 (0.56–1.12) p=0.21
0.82 (0.70–0.97) p=0.021
0.83 (0.73–0.93) p=0.00169
0.82 (0.71–0.95) p=0.00787
0.83 (0.73–0.94) p=0.00245
0.5
0.7
1
1.5
2
OR per 1−unit increase in genetically predicted PDCD5 expression
B
rs78674413
rs115154796
rs6510251
rs118131802
rs4804994
rs140868469
rs146200127
rs140637697
rs145346053
rs149305472
rs78768752
rs145288127
rs79541583
rs259243
rs34363498
rs28521355
All − MR Egger
All − Inverse variance weighted
MR effect size for 'PDCD5 expression (eQTLGen)' on 'PBC (Cordell meta−GWAS)'
C
MR Estimate
Inverse variance weighted (fixed effects)
Inverse variance weighted (multiplicative random effects)
MR Egger
Simple mode
Weighted median
Weighted mode
SNP effect on PBC (Cordell meta−GWAS)
SNP effect on PDCD5 expression (eQTLGen)
D
rs11671790
rs28521355
rs8100642
rs73036017
rs11669589
rs2112420
rs117401331
rs4804994
rs7256841
rs78768752
rs3815760
rs6510288
rs34013687
rs10402931
rs6510251
rs34363498
rs73038273
rs3855674
rs115154796
rs12608785
rs259243
rs4805744
rs78674413
All − MR Egger
All − Inverse variance weighted
MR effect size for 'PDCD5 expression (eQTLGen)' on 'PBC (FinnGen R12)'
E
MR Estimate
Inverse variance weighted (fixed effects)
Inverse variance weighted (multiplicative random effects)
MR Egger
Simple mode
Weighted median
Weighted mode
SNP effect on PBC (FinnGen R12)
SNP effect on PDCD5 expression (eQTLGen)

Figure S2. PDCD5 MR in additional PBC GWAS. (A) Summary estimates; (B–C) GCST90061440 forest and scatter plots; (D–E) FinnGen R12 forest and scatter plots. Random-effects IVW estimates are OR 0.827 (95% CI 0.731–0.935; P = 0.00245) and OR 0.82 (95% CI 0.71–0.95; P = 0.00787), respectively.

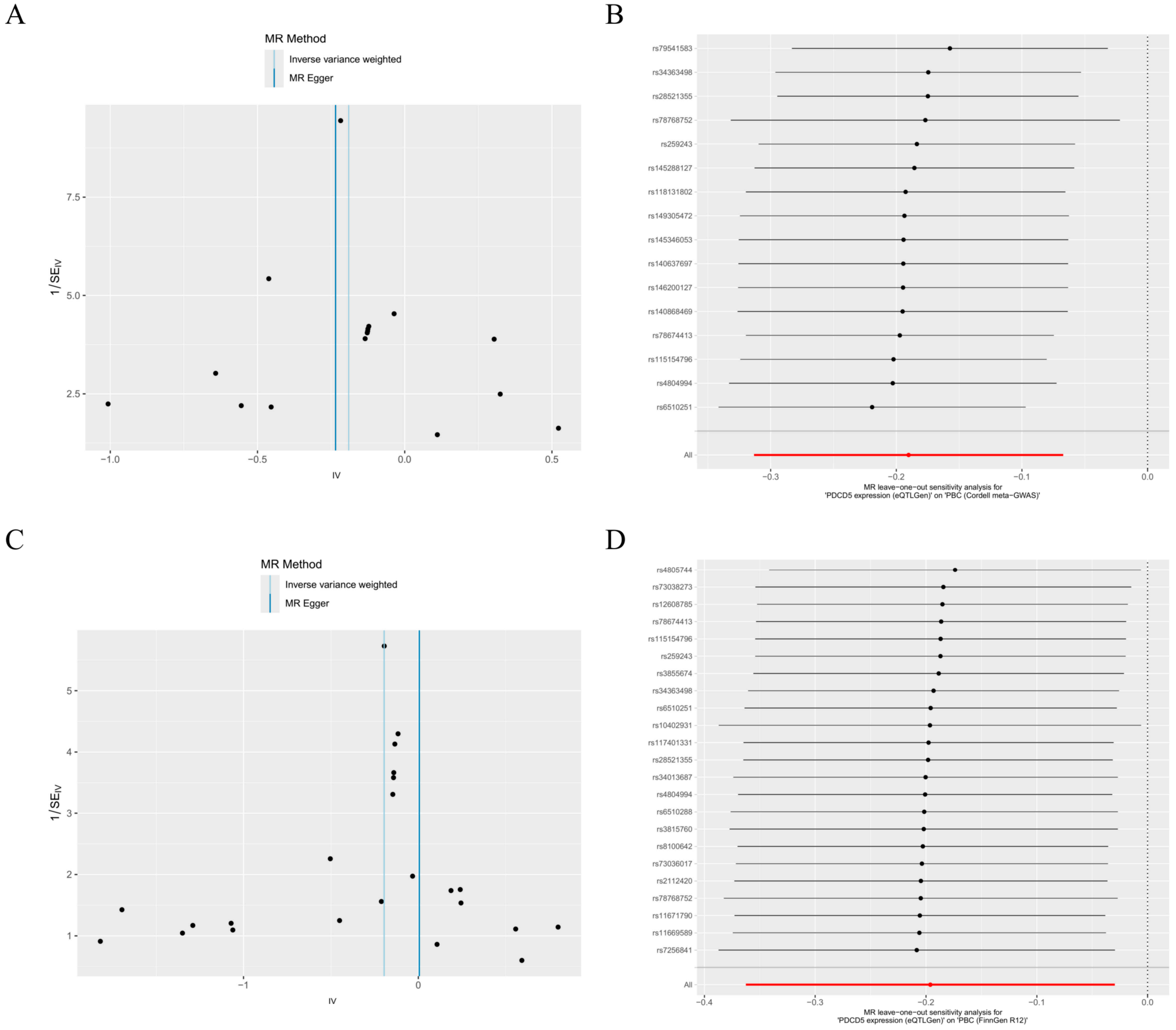

Figure S3. Sensitivity analyses for additional PBC GWAS. (A–B) GCST90061440 funnel and leave-one-out plots. (C–D) FinnGen R12 funnel and leave-one-out plots.

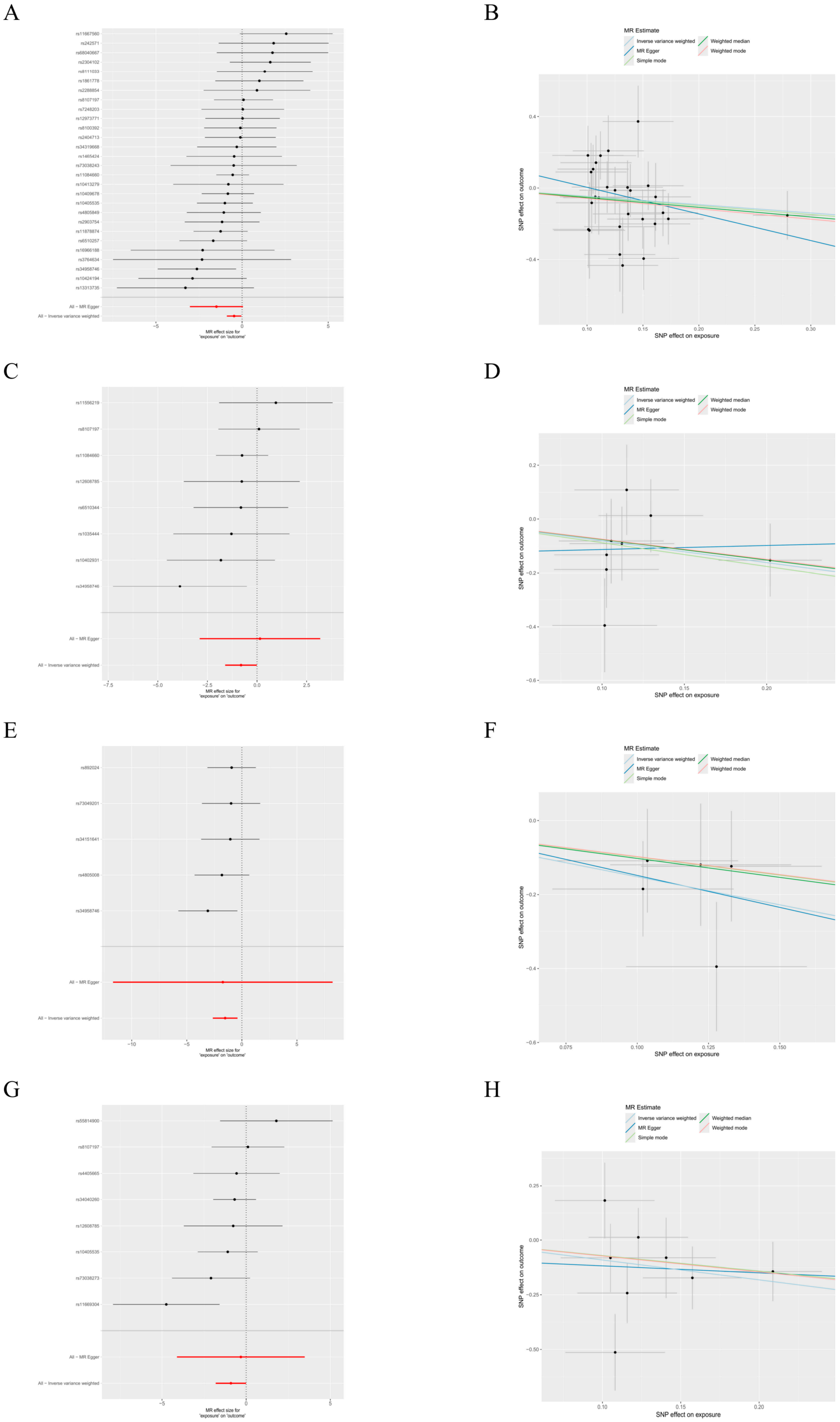
A
B
MR Estimate
Inverse variance weighted
MR Egger
Simple mode
Weighted median
Weighted mode
SNP effect on outcome
SNP effect on exposure
All - MR Egger
All - Inverse variance weighted
MR effect size for 'exposure' on 'outcome'
C
D
E
F
G
H

Figure S4. Immune-cell PDCD5 MR. Forest/scatter pairs are shown for (A–B) CD4 NC, (C–D) CD8 NC, (E–F) dendritic cells and (G–H) natural killer cells.

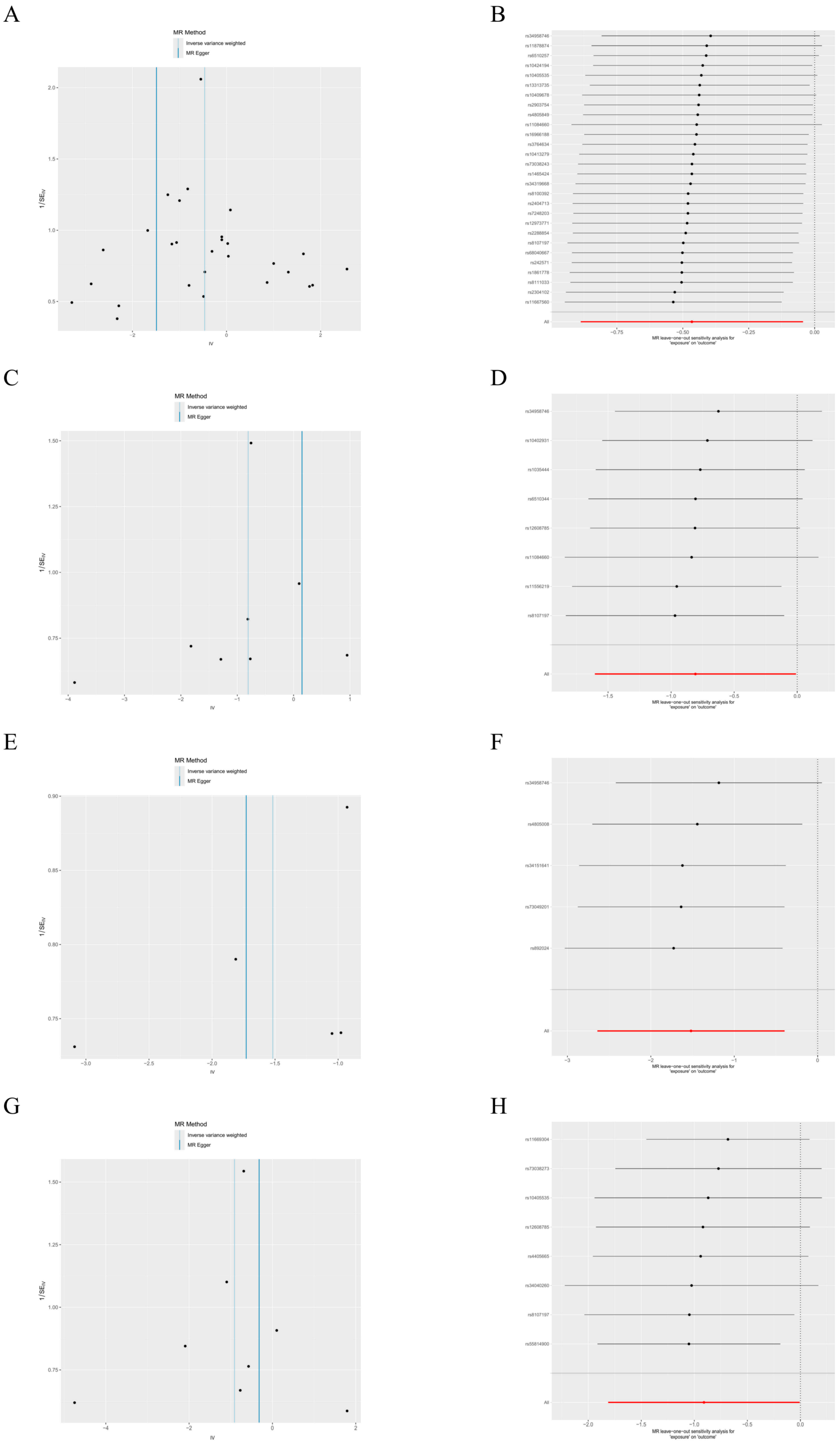
A
B
C
D
E
F
G
H
MR Method
Inverse variance weighted
MR Egger
1/SE$_{IV}$
IV
MR leave-one-out sensitivity analysis for
'exposure' on 'outcome'
All

Figure S5. Immune-cell MR sensitivity analyses. Funnel/leave-one-out pairs are shown for (A–B) CD4 NC, (C–D) CD8 NC, (E–F) dendritic cells and (G–H) natural killer cells.

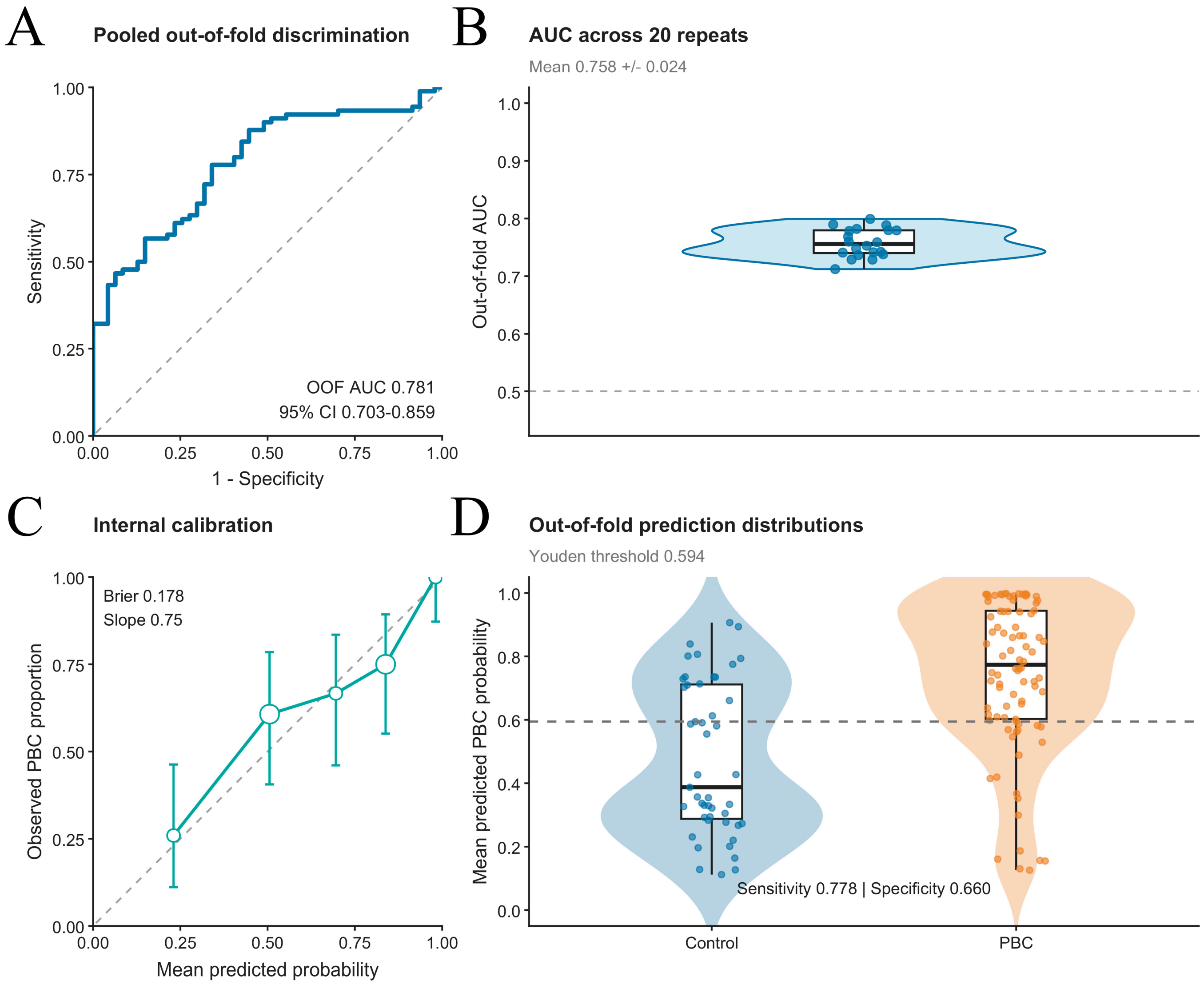

Figure S6. Internal machine-learning validation. (A) ROC curve based on participant-averaged out-of-fold predictions and DeLong 95% CI. (B) AUC across 20 repeated five-fold outer validations. (C) Calibration plot with binomial 95% intervals. (D) Prediction distributions and the Youden threshold. The analysis evaluates the downstream model conditional on the eight preselected candidate genes.
