# Supplementary material for: Integrated Multi-Omics and Experimental Evidence Implicates PDCD5 as a Candidate Gene Associated with Primary Biliary Cholangitis
Source: Life (Basel). 2026 Sep 22;16(10):1583. doi: 10.3390/life16101583 (PMC13705998; doi:10.3390/life16101583)
Supplement: Supplementary file 1 [file life-16-01583-s001.zip › Supplementary_Computational_Files.zip!/Supplementary_Computational_Files/Computational_Files/Supplementary_Figure_ML_Internal_Validation.pdf]

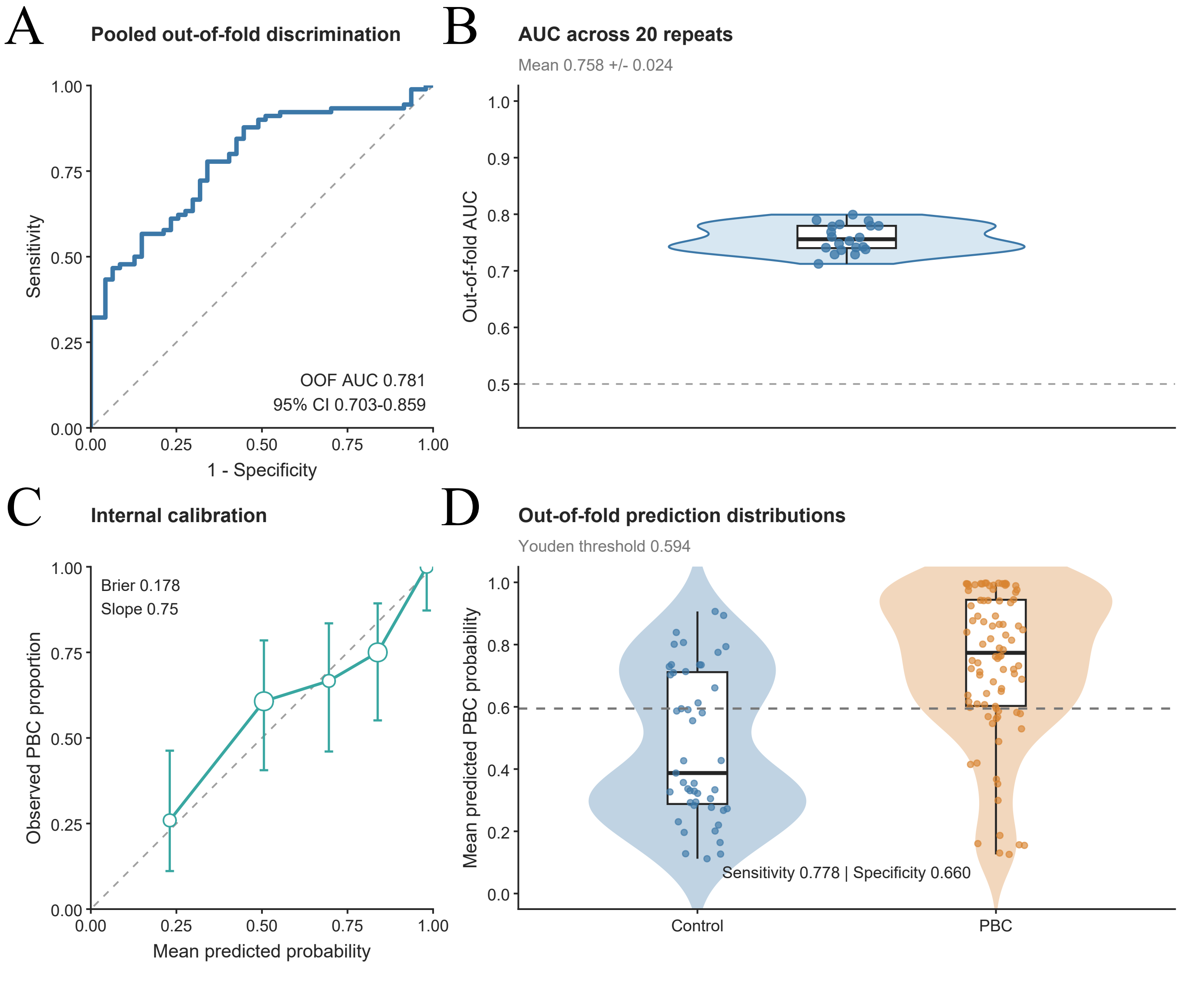
A
Pooled out-of-fold discrimination
Sensitivity
1 - Specificity
OOF AUC 0.781
95% CI 0.703-0.859
B
AUC across 20 repeats
Mean 0.758 +/- 0.024
Out-of-fold AUC
C
Internal calibration
Brier 0.178
Slope 0.75
Observed PBC proportion
Mean predicted probability
D
Out-of-fold prediction distributions
Youden threshold 0.594
Mean predicted PBC probability
Sensitivity 0.778 | Specificity 0.660
Control
PBC
