# Supplementary material for: Integrated Multi-Omics and Experimental Evidence Implicates PDCD5 as a Candidate Gene Associated with Primary Biliary Cholangitis
Source: Life (Basel). 2026 Sep 22;16(10):1583. doi: 10.3390/life16101583 (PMC13705998; doi:10.3390/life16101583)
Supplement: Supplementary file 1 [file life-16-01583-s001.zip › Table_S6_Primary_MR_Outputs.zip!/AKT1/forest.pdf]

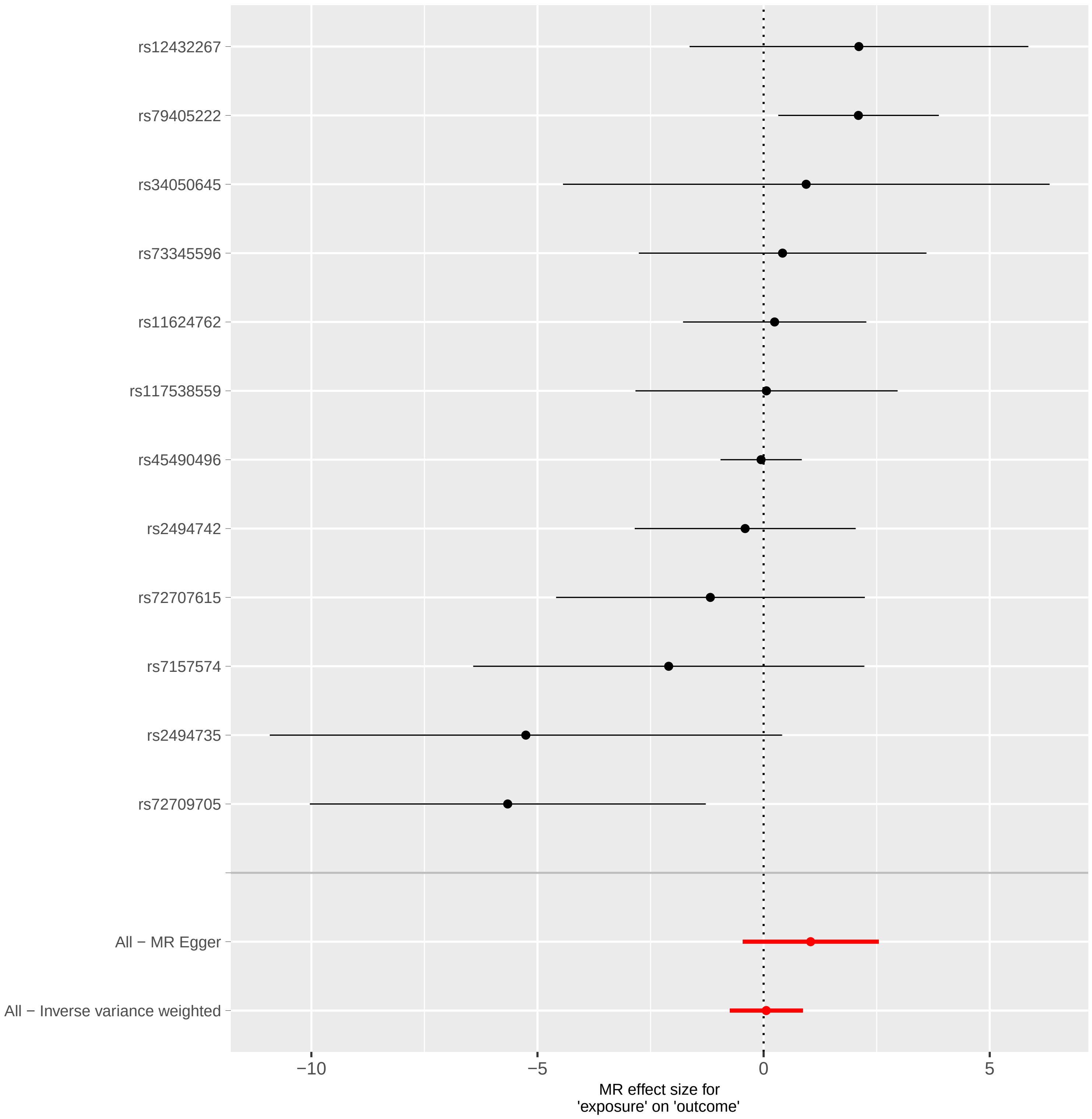

rs12432267
rs79405222
rs34050645
rs73345596
rs11624762
rs117538559
rs45490496
rs2494742
rs72707615
rs7157574
rs2494735
rs72709705
All − MR Egger
All − Inverse variance weighted
−10
−5
0
5
MR effect size for
'exposure' on 'outcome'
