# Supplementary material for: Integrated Multi-Omics and Experimental Evidence Implicates PDCD5 as a Candidate Gene Associated with Primary Biliary Cholangitis
Source: Life (Basel). 2026 Sep 22;16(10):1583. doi: 10.3390/life16101583 (PMC13705998; doi:10.3390/life16101583)
Supplement: Supplementary file 1 [file life-16-01583-s001.zip › Table_S6_Primary_MR_Outputs.zip!/Forest plot.pdf]

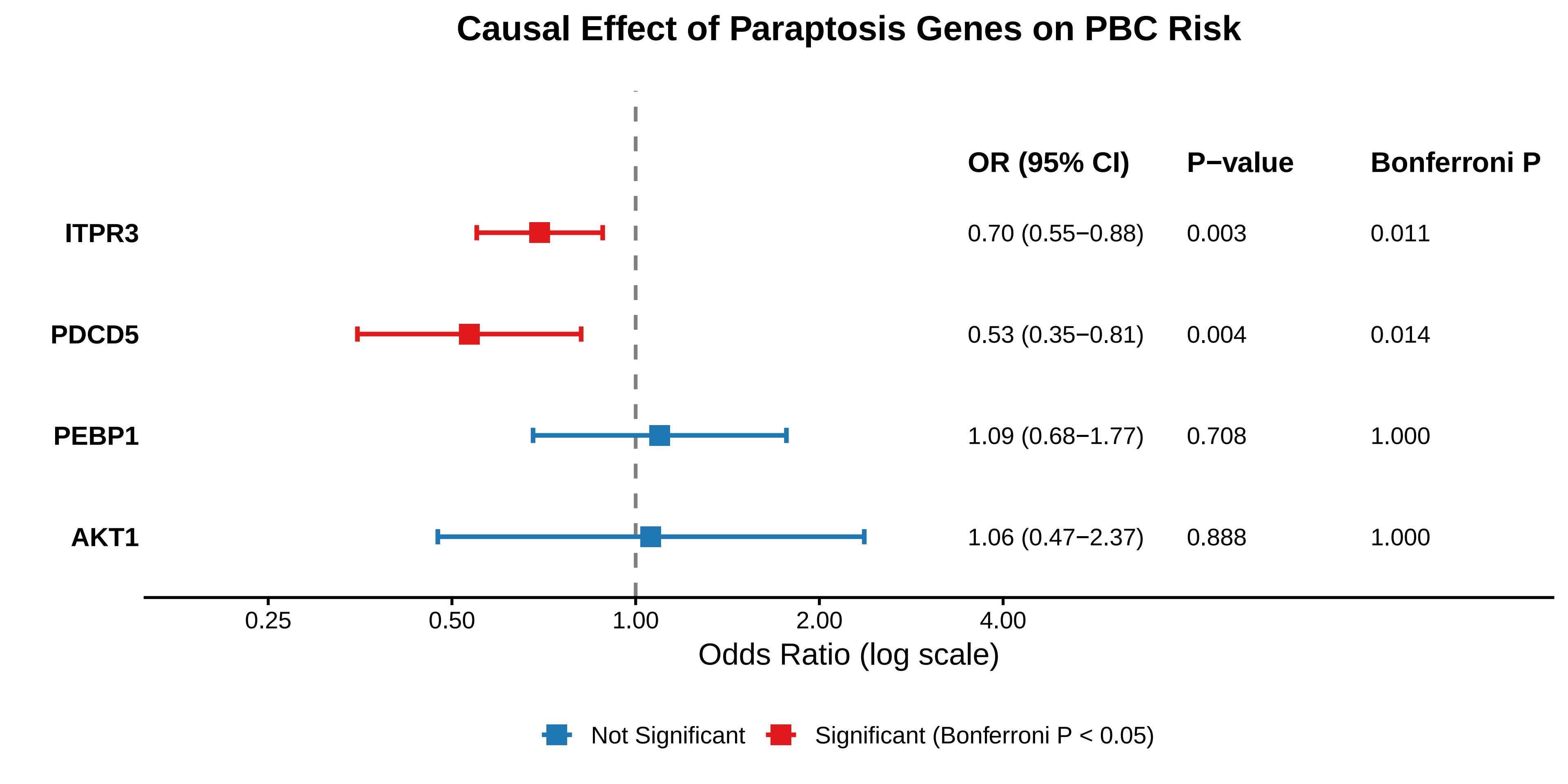
Causal Effect of Paraptosis Genes on PBC Risk
OR (95% CI)
P−value
Bonferroni P
ITPR3
0.70 (0.55−0.88)
0.003
0.011
PDCD5
0.53 (0.35−0.81)
0.004
0.014
PEBP1
1.09 (0.68−1.77)
0.708
1.000
AKT1
1.06 (0.47−2.37)
0.888
1.000
0.25
0.50
1.00
2.00
4.00
Odds Ratio (log scale)
Not Significant
Significant (Bonferroni P < 0.05)
