# Supplementary material for: Integrated Multi-Omics and Experimental Evidence Implicates PDCD5 as a Candidate Gene Associated with Primary Biliary Cholangitis
Source: Life (Basel). 2026 Sep 22;16(10):1583. doi: 10.3390/life16101583 (PMC13705998; doi:10.3390/life16101583)
Supplement: Supplementary file 1 [file life-16-01583-s001.zip › Table_S6_Primary_MR_Outputs.zip!/ITPR3/forest.pdf]

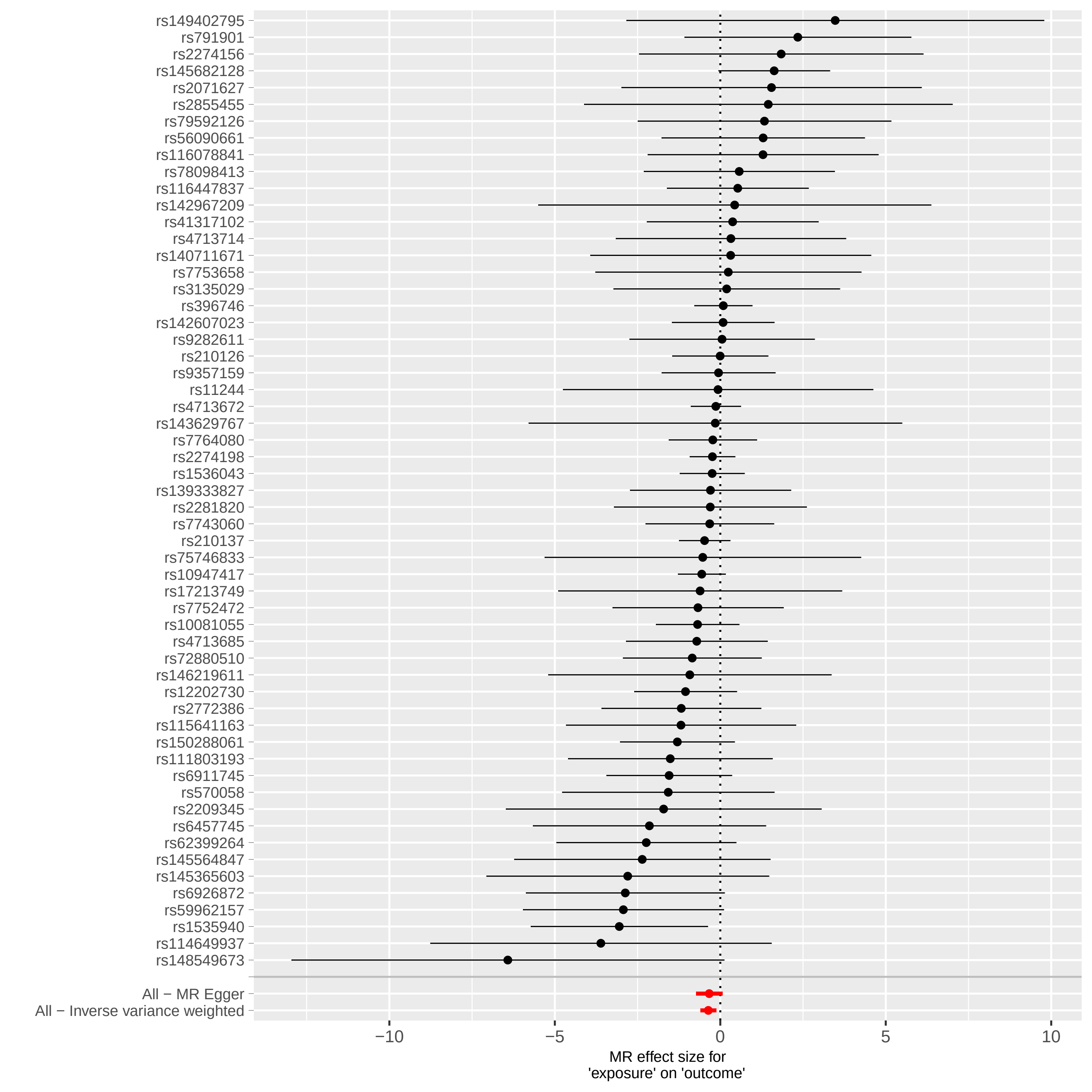
rs149402795
rs791901
rs2274156
rs145682128
rs2071627
rs2855455
rs79592126
rs56090661
rs116078841
rs78098413
rs116447837
rs142967209
rs41317102
rs4713714
rs140711671
rs7753658
rs3135029
rs396746
rs142607023
rs9282611
rs210126
rs9357159
rs11244
rs4713672
rs143629767
rs7764080
rs2274198
rs1536043
rs139333827
rs2281820
rs7743060
rs210137
rs75746833
rs10947417
rs17213749
rs7752472
rs10081055
rs4713685
rs72880510
rs146219611
rs12202730
rs2772386
rs115641163
rs150288061
rs111803193
rs6911745
rs570058
rs2209345
rs6457745
rs62399264
rs145564847
rs145365603
rs6926872
rs59962157
rs1535940
rs114649937
rs148549673
All − MR Egger
All − Inverse variance weighted
−10
−5
0
5
10
MR effect size for
'exposure' on 'outcome'
