# Supplementary material for: Integrated Multi-Omics and Experimental Evidence Implicates PDCD5 as a Candidate Gene Associated with Primary Biliary Cholangitis
Source: Life (Basel). 2026 Sep 22;16(10):1583. doi: 10.3390/life16101583 (PMC13705998; doi:10.3390/life16101583)
Supplement: Supplementary file 1 [file life-16-01583-s001.zip › Table_S6_Primary_MR_Outputs.zip!/ITPR3/funnelplot.pdf]

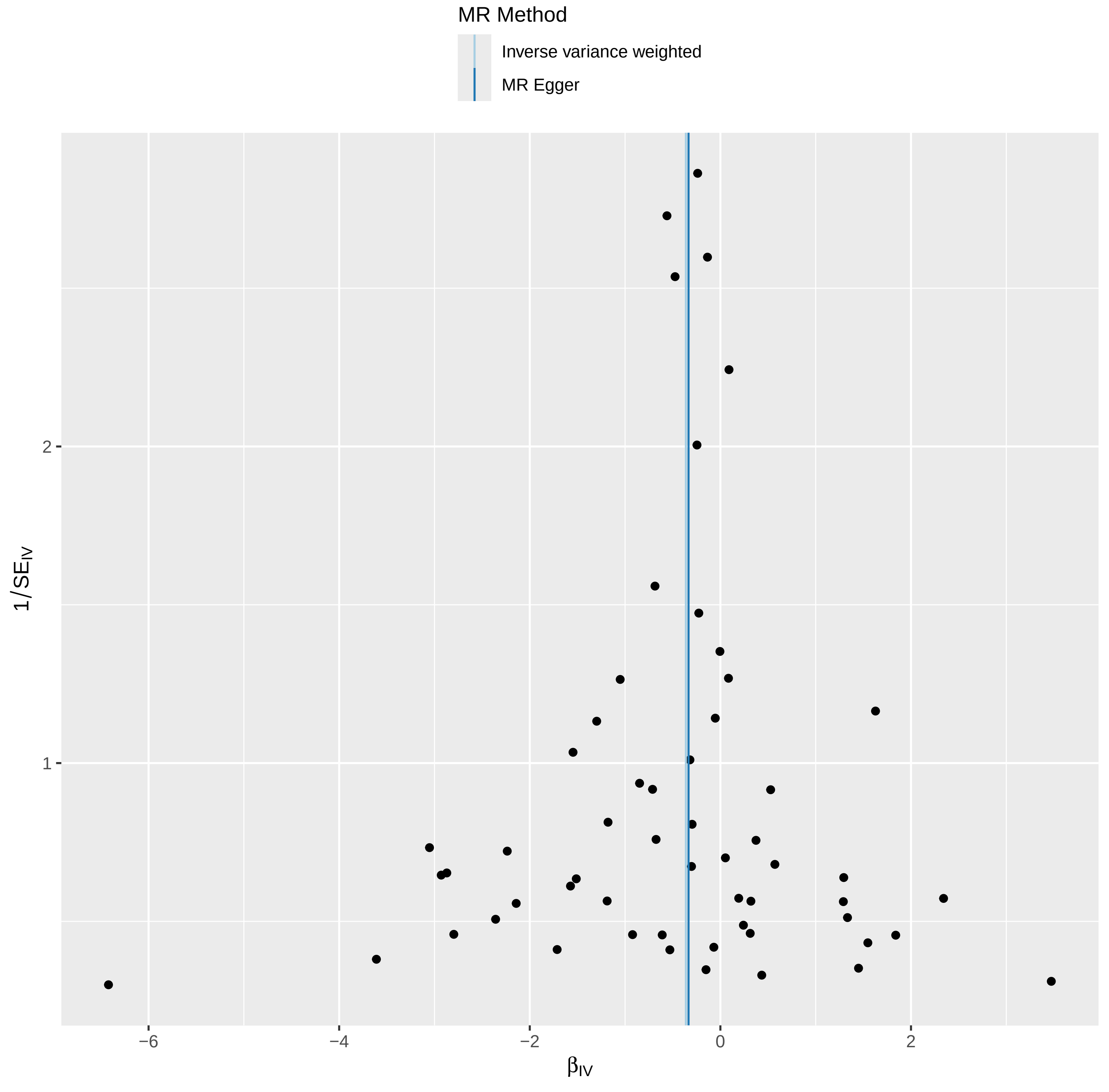

MR Method
Inverse variance weighted
MR Egger
1/SE$_{IV}$
2
1
−6
−4
−2
0
2
β$_{IV}$
