# Supplementary material for: Integrated Multi-Omics and Experimental Evidence Implicates PDCD5 as a Candidate Gene Associated with Primary Biliary Cholangitis
Source: Life (Basel). 2026 Sep 22;16(10):1583. doi: 10.3390/life16101583 (PMC13705998; doi:10.3390/life16101583)
Supplement: Supplementary file 1 [file life-16-01583-s001.zip › Table_S6_Primary_MR_Outputs.zip!/ITPR3/sensitivity-analysis.pdf]

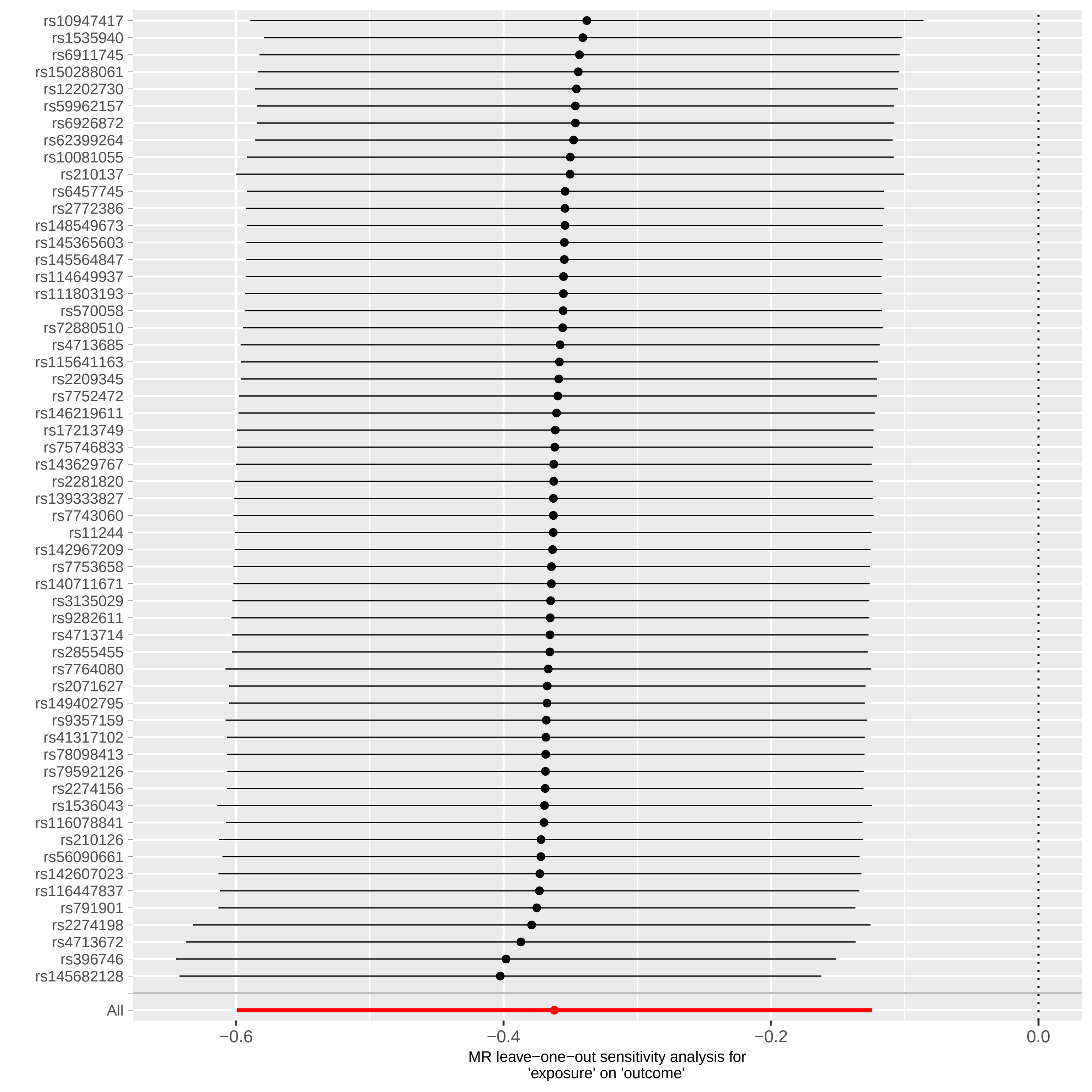
rs10947417
rs1535940
rs6911745
rs150288061
rs12202730
rs59962157
rs6926872
rs62399264
rs10081055
rs210137
rs6457745
rs2772386
rs148549673
rs145365603
rs145564847
rs114649937
rs111803193
rs570058
rs72880510
rs4713685
rs115641163
rs2209345
rs7752472
rs146219611
rs17213749
rs75746833
rs143629767
rs2281820
rs139333827
rs7743060
rs11244
rs142967209
rs7753658
rs140711671
rs3135029
rs9282611
rs4713714
rs2855455
rs7764080
rs2071627
rs149402795
rs9357159
rs41317102
rs78098413
rs79592126
rs2274156
rs1536043
rs116078841
rs210126
rs56090661
rs142607023
rs116447837
rs791901
rs2274198
rs4713672
rs396746
rs145682128
All
−0.6
−0.4
−0.2
0.0
MR leave−one−out sensitivity analysis for
'exposure' on 'outcome'
