# Supplementary material for: Integrated Multi-Omics and Experimental Evidence Implicates PDCD5 as a Candidate Gene Associated with Primary Biliary Cholangitis
Source: Life (Basel). 2026 Sep 22;16(10):1583. doi: 10.3390/life16101583 (PMC13705998; doi:10.3390/life16101583)
Supplement: Supplementary file 1 [file life-16-01583-s001.zip › Table_S6_Primary_MR_Outputs.zip!/PDCD5/forest.pdf]

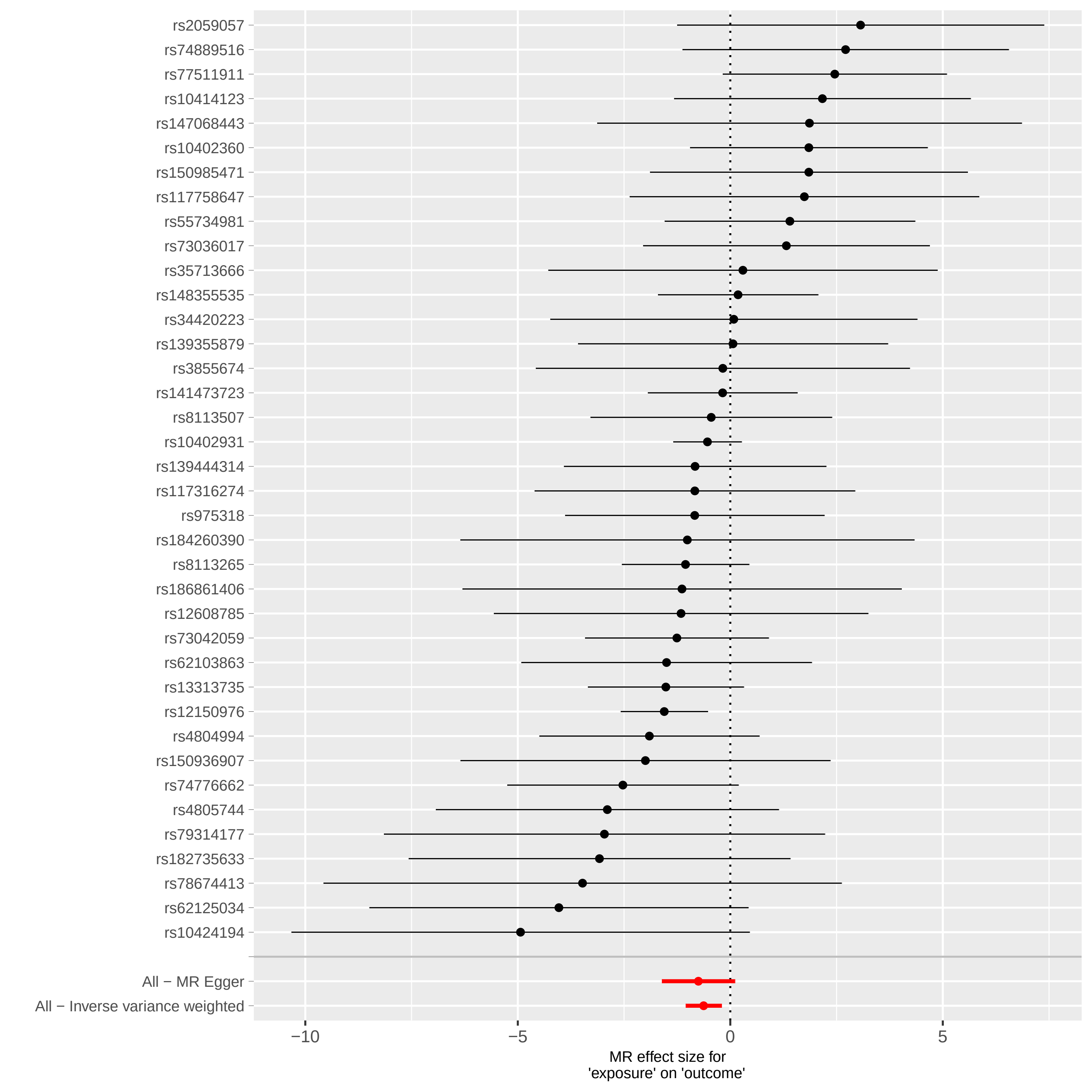
rs2059057
rs74889516
rs77511911
rs10414123
rs147068443
rs10402360
rs150985471
rs117758647
rs55734981
rs73036017
rs35713666
rs148355535
rs34420223
rs139355879
rs3855674
rs141473723
rs8113507
rs10402931
rs139444314
rs117316274
rs975318
rs184260390
rs8113265
rs186861406
rs12608785
rs73042059
rs62103863
rs13313735
rs12150976
rs4804994
rs150936907
rs74776662
rs4805744
rs79314177
rs182735633
rs78674413
rs62125034
rs10424194
All − MR Egger
All − Inverse variance weighted
−10
−5
0
5
MR effect size for
'exposure' on 'outcome'
