# Supplementary material for: Integrated Multi-Omics and Experimental Evidence Implicates PDCD5 as a Candidate Gene Associated with Primary Biliary Cholangitis
Source: Life (Basel). 2026 Sep 22;16(10):1583. doi: 10.3390/life16101583 (PMC13705998; doi:10.3390/life16101583)
Supplement: Supplementary file 1 [file life-16-01583-s001.zip › Table_S6_Primary_MR_Outputs.zip!/PDCD5/sensitivity-analysis.pdf]

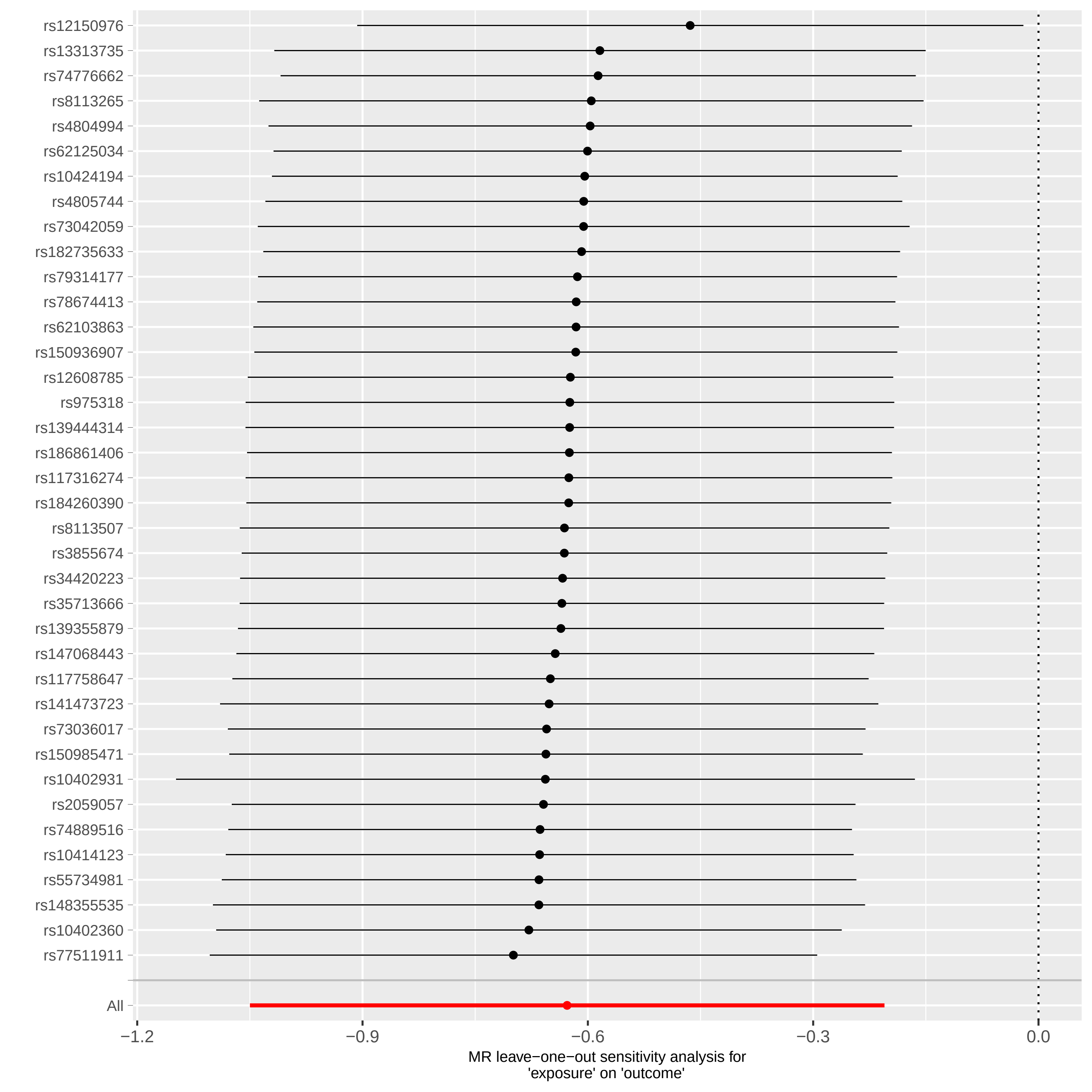
rs12150976
rs13313735
rs74776662
rs8113265
rs4804994
rs62125034
rs10424194
rs4805744
rs73042059
rs182735633
rs79314177
rs78674413
rs62103863
rs150936907
rs12608785
rs975318
rs139444314
rs186861406
rs117316274
rs184260390
rs8113507
rs3855674
rs34420223
rs35713666
rs139355879
rs147068443
rs117758647
rs141473723
rs73036017
rs150985471
rs10402931
rs2059057
rs74889516
rs10414123
rs55734981
rs148355535
rs10402360
rs77511911
All
−1.2
−0.9
−0.6
−0.3
0.0
MR leave−one−out sensitivity analysis for
'exposure' on 'outcome'
