# Supplementary material for: Integrated Multi-Omics and Experimental Evidence Implicates PDCD5 as a Candidate Gene Associated with Primary Biliary Cholangitis
Source: Life (Basel). 2026 Sep 22;16(10):1583. doi: 10.3390/life16101583 (PMC13705998; doi:10.3390/life16101583)
Supplement: Supplementary file 1 [file life-16-01583-s001.zip › Table_S6_Primary_MR_Outputs.zip!/PEBP1/forest.pdf]

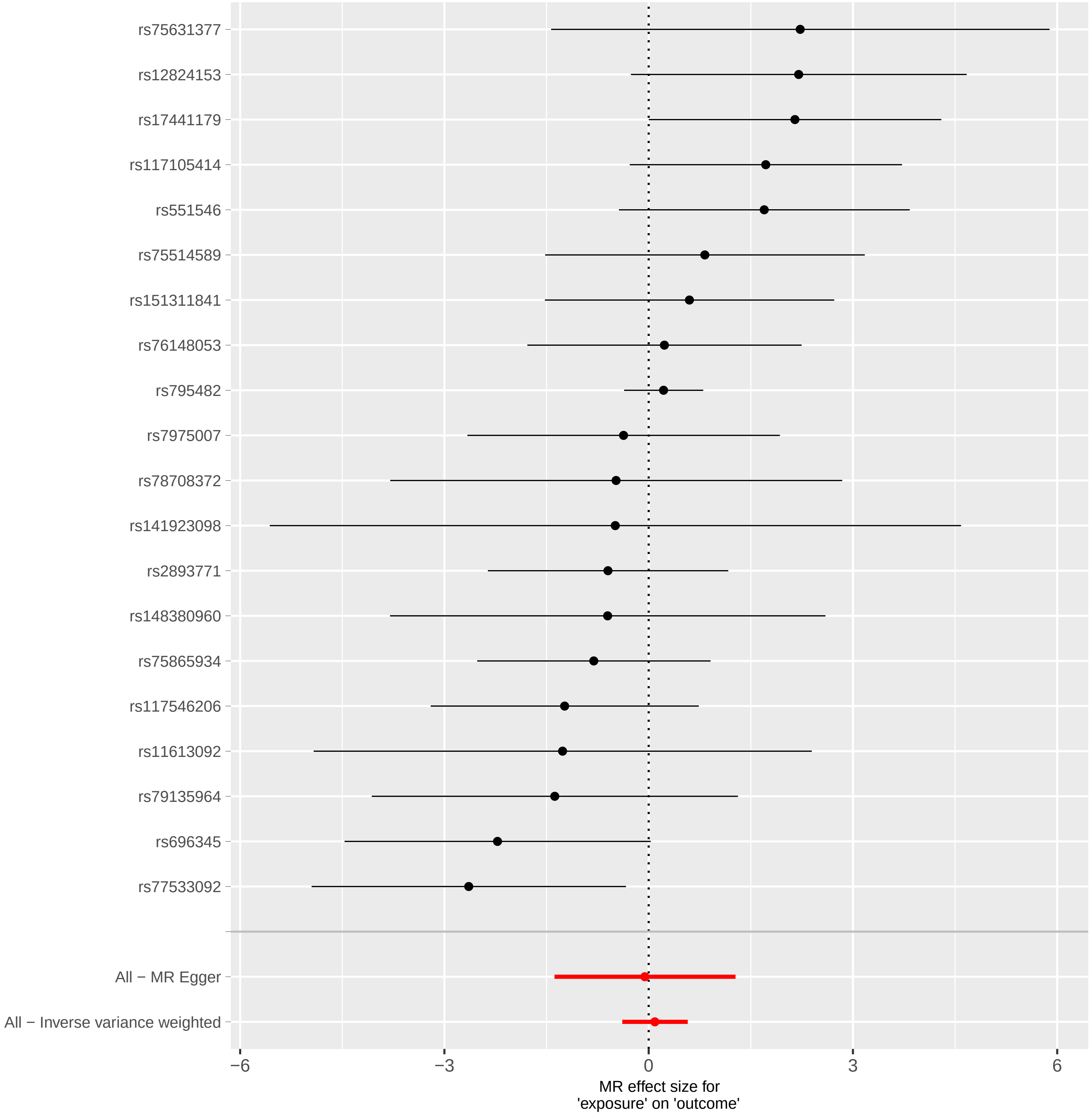

rs75631377
rs12824153
rs17441179
rs117105414
rs551546
rs75514589
rs151311841
rs76148053
rs795482
rs7975007
rs78708372
rs141923098
rs2893771
rs148380960
rs75865934
rs117546206
rs11613092
rs79135964
rs696345
rs77533092
All − MR Egger
All − Inverse variance weighted
−6
−3
0
3
6
MR effect size for
'exposure' on 'outcome'
