# Supplementary material for: Integrated Multi-Omics and Experimental Evidence Implicates PDCD5 as a Candidate Gene Associated with Primary Biliary Cholangitis
Source: Life (Basel). 2026 Sep 22;16(10):1583. doi: 10.3390/life16101583 (PMC13705998; doi:10.3390/life16101583)
Supplement: Supplementary file 1 [file life-16-01583-s001.zip › Table_S6_Primary_MR_Outputs.zip!/PEBP1/sensitivity-analysis.pdf]

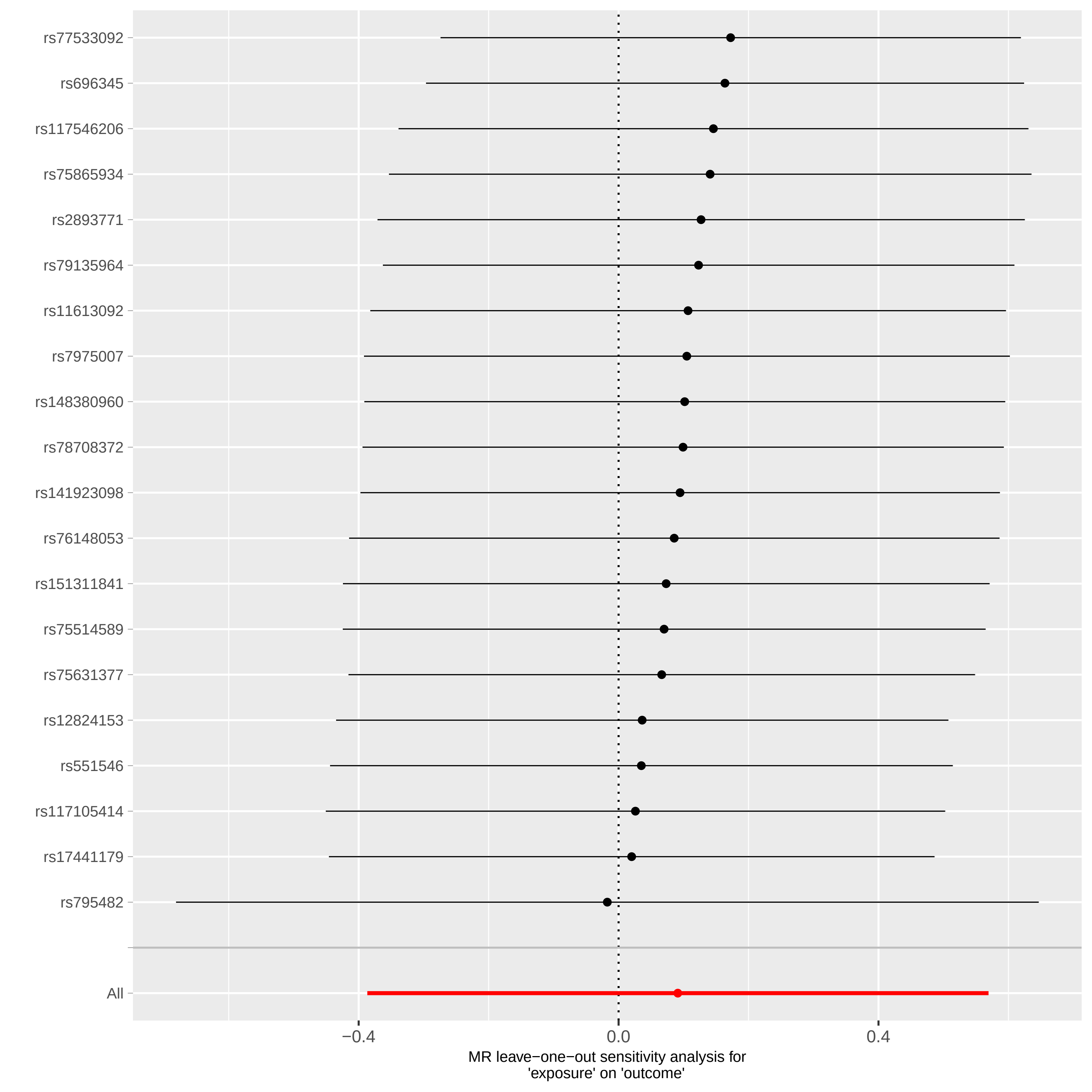
rs77533092
rs696345
rs117546206
rs75865934
rs2893771
rs79135964
rs11613092
rs7975007
rs148380960
rs78708372
rs141923098
rs76148053
rs151311841
rs75514589
rs75631377
rs12824153
rs551546
rs117105414
rs17441179
rs795482
All
−0.4
0.0
0.4
MR leave−one−out sensitivity analysis for
'exposure' on 'outcome'
